# Phospholipid Signaling in Crop Plants: A Field to Explore

**DOI:** 10.3390/plants13111532

**Published:** 2024-05-31

**Authors:** Lucas Amokrane, Igor Pokotylo, Sébastien Acket, Amélie Ducloy, Adrian Troncoso-Ponce, Jean-Luc Cacas, Eric Ruelland

**Affiliations:** 1 Unité Génie Enzymatique & Cellulaire, Université de Technologie de Compiègne, UMR CNRS 7025, 60200 Compiègne, France; lucas.amokrane@utc.fr (L.A.); igor.pokotylo@agroparistech.fr (I.P.); sebastien.acket@utc.fr (S.A.); adrian.troncoso-ponce@utc.fr (A.T.-P.); 2 INRAE, AgroParisTech, Institute Jean-Pierre Bourgin (IJPB), University Paris-Saclay, 78000 Versailles, Francejean-luc.cacas@inrae.fr (J.-L.C.)

**Keywords:** crop species, lipid signaling, phosphatidic acid, phospholipase, diacylglycerol kinase, environmental stresses, signaling, protein structure

## Abstract

In plant models such as *Arabidopsis thaliana*, phosphatidic acid (PA), a key molecule of lipid signaling, was shown not only to be involved in stress responses, but also in plant development and nutrition. In this article, we highlight lipid signaling existing in crop species. Based on open access databases, we update the list of sequences encoding phospholipases D, phosphoinositide-dependent phospholipases C, and diacylglycerol-kinases, enzymes that lead to the production of PA. We show that structural features of these enzymes from model plants are conserved in equivalent proteins from selected crop species. We then present an in-depth discussion of the structural characteristics of these proteins before focusing on PA binding proteins. For the purpose of this article, we consider RESPIRATORY BURST OXIDASE HOMOLOGUEs (RBOHs), the most documented PA target proteins. Finally, we present pioneering experiments that show, by different approaches such as monitoring of gene expression, use of pharmacological agents, ectopic over-expression of genes, and the creation of silenced mutants, that lipid signaling plays major roles in crop species. Finally, we present major open questions that require attention since we have only a perception of the peak of the iceberg when it comes to the exciting field of phospholipid signaling in plants.

## 1. Introduction

**Figure 1:**
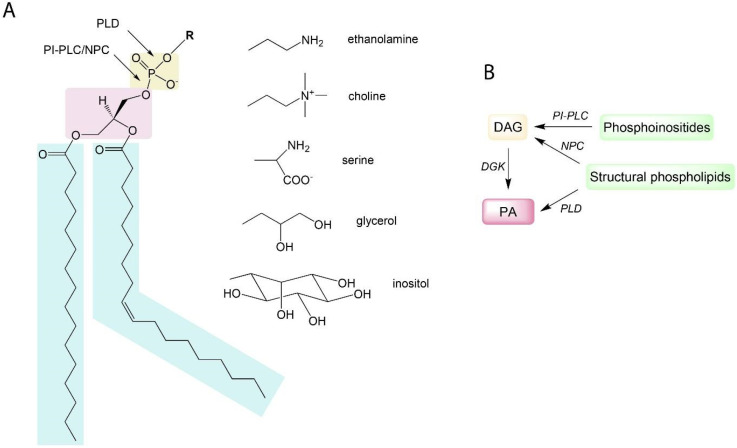
Schematic representation of phosphoglycerolipid structure and pathways leading to PA production in plants: (**A**) A phosphoglycerolipid structure with indicated sites of hydrolysis by phospholipases C and D. Light blue boxes: fatty acids; pink box: glycerol; brown box: phosphate. R is for polar head group. Fatty acid composition and desaturation level of a phosphoglycerolipid can differ. Shown here is what is known as “16:0–18:1 phosphoglycerolipid” containing a saturated fatty acid with a 16-carbon chain in the *sn*-1 position and a monounsaturated fatty acid with an 18-carbon chain in the *sn*-2 position. (**B**) A simplified scheme of pathways leading to PA production. PLD, phospholipase D; NPC, non-specific phospholipase; PI-PLC, phosphoinositide-dependent phospholipases C; DGK, diacylglycerol kinase; DAG, diacylglycerol.

Phosphoglycerolipids are lipids consisting of a diacyglycerol (DAG) backbone linked to a polar head through a phosphodiester link. Polar heads can be choline, glycerol, ethanolamine, inositol, or serine giving rise to phosphatidylcholine (PC), phosphatidylglycerol (PG), phosphatidylethanolamine (PE), phosphatidylinositol (PI), and phosphatidylserine (PS) (Figure 1A). Phosphatidic acid (PA) is the simplest phosphoglycerolipid, being a phosphorylated DAG. Galactolipids consist of a DAG backbone esterified with one or more (up to four) galactosyl residues. Galactolipids and phospholipids, but also triacylglycerols, are glycerolipids.

Phospholipids and/or galactolipids are key constituents of biological membranes [1]. In plasma membranes of *Arabidopsis thaliana* leaves, phosphoglycerolipids (or simply called phospholipids) account for 47% of total lipid content with sterols taking up another 46% and sphingolipids are measured at 7% [2]. In more recent studies that take into account such sphingolipid classes as glycosyl inositol phosphorylceramides, it was demonstrated that the share of sphingolipids in plasma membranes could actually be much higher, up to 47 mol% in plasma membranes of tobacco leaves [3]. In other types of plant cell membranes, the lipid composition could be drastically different. Membranes of chloroplasts primarily consist of galactolipids (80%) [4]. The nature of the fatty acids esterified to the glycerol in glycerolipids influences the fluidity of the membrane. The higher the desaturation, the more fluid the membrane [5]. In concert with proteins (membrane-bound enzymes and transport proteins), glycerolipids form selectively permeable barriers that delineate the interfaces between individual cell compartments. Membranes act specifically as a cell–environment interface and are the primary site for signaling events leading to plant acclimation to changing environment conditions. For instance, cold stress is perceived through membrane rigidification [6] and osmotic stress can be perceived through membrane-bound histidine kinase [7,8]. Plant hormones, like ethylene, cytokinins, and brassinosteroids are perceived by transmembrane receptors, with brassinosteroids receptors located specifically on the plasma membrane [9]. The so-called Pathogen Associated Molecular Patterns (PAMPs)—which are molecular motifs associated with pathogens—are perceived by Pattern Recognition Receptors (PRR)—transmembrane receptors located on the plasma membrane, such as FLS2 receptor of flagellin [10]. Membrane lipids would thus have a direct impact on membrane-bound receptor proteins. In animals, phosphatidylinositol-4,5-bisphosphate (PI-4,5-P_2_) was shown to promote the internalization of β2 adrenergic receptor from the plasma membrane to early endosomes [11]. In organelles like chloroplasts and mitochondria, membranes host important energy metabolism activities such as the electron transport chains for photosynthesis and respiration, respectively. Chloroplast membranes are also known to undergo stress-induced remodeling as a part of plant acclimation to stresses [12].

Besides their structural functions, phospholipids also have a signaling role. In plants, lipid second messengers include hydrophobic phospholipid-derivatives such as PA and lysophospholipids. Phosphoinositides, minor phospholipids that possess an inositol head group phosphorylated in several positions, are also signaling lipids [13], and they include phosphatidylinositol-4-phosphate (PI-4-P) and phosphatidylinositol-4,5-bisphosphate (PI-4,5-P_2_). Signaling lipids in plants are the products of phospholipases, lipid kinases, or lipid phosphatases [14]. They can be rapidly accumulated (within minutes) in plant membranes in response to various stimuli. For instance, PA levels increase after a 5 min exposure to a chilling stress in Arabidopsis suspension cells [15] or when Arabidopsis roots are salt stressed [16]. Signaling lipids can bind/tether cytosolic proteins, thus affecting their localization and/or activity. In Arabidopsis, PA directly binds and activates RBOHD [17,18]. Signaling lipids can also alter membrane dynamics, thus impacting vesicle trafficking, endocytosis, or other modes of transmembrane transport. For instance, PA has a cone shape with a small polar head group and, therefore, its local accumulation favors a negative membrane curvature and thus stimulates endocytosis [19]. 

During past decades, PA has emerged as a major plant signaling molecule. It is produced either via the hydrolytic cleavage of phospholipids by PLD or by DAG phosphorylation by DGK (Figure 1A,B). DAG can be produced from the action of phospholipases C (PLC). Some PLCs act specifically on phosphoinositides, the so-called phosphoinositide-dependent phospholipases C (PI-PLC) [20]; alternatively, non-specific phospholipases C (NPC) can act on structural phospholipids such as phosphatidylcholine (PC) or phosphatidylethanolamine (PE) [21,22]. Interestingly, the length and desaturation level of fatty acids (FA) esterified to PE, PC, or PI-4,5-P_2_ phospholipids are not identical. For example, polyphosphoinositides (including PI-4,5-P_2_), unlike PE or PC, were shown to contain mostly saturated FA in plasma membranes from tobacco leaves [23]. Inherently, PA produced via alternative pathways will not be the same from a molecular point of view. Divergent ‘molecular species’ of PA will thus be produced; this has an impact on PA functionality and a set of intracellular targets [14]. In Arabidopsis, PA has been shown to be involved in responses to a plethora of abiotic stresses, including cold, drought, heat, etc. [14,24]. This is probably associated with the fact that PA is accumulated in response to hormones, such as abscisic acid (ABA) and salicylic acid (SA) [25,26]. PA also transduces the response of signaling peptides, including PAMPs such as cryptogein [27] or flagellin [28] and phytocytokines such as SCOOP peptides [29]. In plants, dozens of PA-binding proteins having different physiological roles have been identified [30,31]. In Arabidopsis, PLDδ-produced PA mediates glyceraldehyde-3-phosphate dehydrogenase (GAPDH) translocation to the nucleus in response to heat stress acclimation [32]. 

Recently, PLDα1-derived PA was shown to be involved in virus resistance mechanisms in tobacco by binding and stimulating the activity of WIPK, SIPK, and NTF4 mitogen-activated protein kinases (MAPKs) [33]. To date, no consensus PA-binding domain has been established in proteins. However, PA binding to proteins is typically ensured by positively charged residues such as arginine and lysine because the phosphate head group of PA has a negative charge at a physiological pH. In some proteins, PA binding can be pinpointed to a specific arginine residue [34]. The contribution of hydrophobic residues to an interaction with the hydrophobic tail of PA is known for some animal and yeast PA binding proteins, but this is yet to be confirmed in plants [14,35]. 

Over the last decades, plant models have yielded an important amount of data, allowing the discovery of some of the molecular mechanisms of lipid signaling such as the role of PA in regulating the Salt Overly Sensitive (SOS) pathway involved in salt stress resistance [36]. The early sequencing of the Arabidopsis genome, the availability of different Arabidopsis mutant collections, its short life cycle and easy genetic transformation have allowed breakthrough advances in deciphering the importance of lipid signaling and its role in plant stress acclimation. So, confronted with an ever-increasing human population, global warming, and climate change, it has become important to assess the presence and roles of lipid signaling in economically important crop species. In this article, we will show that enzymes of the lipid signaling pathways are present in crop plants and that structural features identified in model plants are conserved in equivalent crop proteins. An in-depth discussion of protein structural characteristics will be illustrated using structural models of proteins obtained using AlphaFold2 [37]. In crops, lipids are mostly considered for their value as energy-storage molecules represented by triacylglycerols. Yet, some phospholipids play important roles in cell signaling and are necessary for environmental stress acclimation. Here, we will consider the lipid signaling pathways that lead to the production of PA and focus on their presence in economically important crop species such as rice, maize, rapeseed, soybean, wheat, sorghum, and potato.

## 2. Results

### 2.1. Enzymes of the Lipid Signaling Pathways in Crop Plants

#### 2.1.1. Phospholipases D

**Figure 2:**
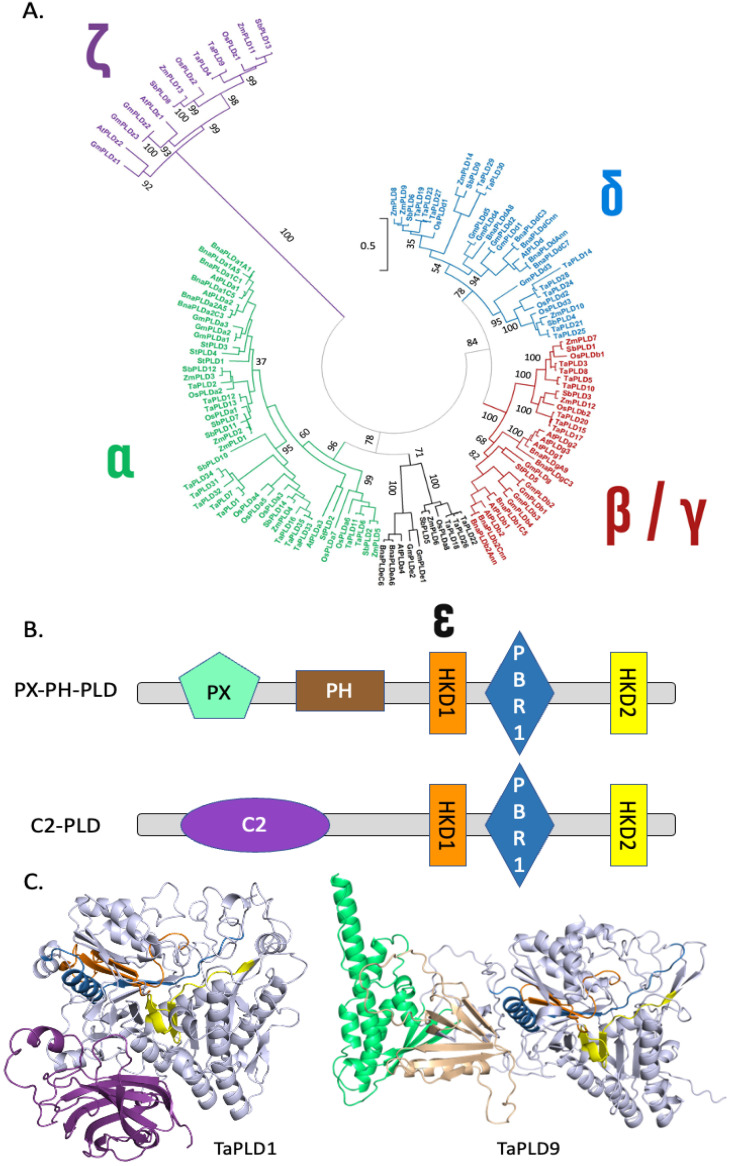
Structural features of the PLD family in plants: (**A**) Phylogenetic tree showing the different PLDs. The accession numbers of the protein sequences used can be found in Table S1. The scale bar refers to a phylogenetic distance that is the average number of substitutions per site (here 0.5). Numbers on the branches indicate bootstrap percentage after 1000 replications in constructing the tree. (**B**) Schematic representation of the structural domains in plant PLDs. Purple, C2 domain; green, PX domain; brown, PH domain; orange, first HKD domain; yellow, second HKD domain; red, PIP2 binding region 1 (PBR1 domain [44]). (**C**) Structural differences between C2-PLDs and PX-PH-PLDs. TaPLD1, a wheat (*Triticum aestivum*) PLD of the α-subtype, was chosen as representative of C2-PLDs, while TaPLD9, a PLD of the *ζ*-subtype, was chosen as representative of PX-PH-PLDs. Note that for TaPLD9, non-structured parts of the protein were represented here. A full representation can be found as Figure S1. The color code used is the same as in (**B**). The Alphafold2 structures can also be colored based on the confidence with which each domain is modeled (Figure S5).

Genes encoding PLDs have been found in all plant genomes sequenced so far including *Oryza sativa* [38], *Zea mays* [39], *Brassica napus* [40], and *Glycine max* [41]. It was possible to retrieve 18, 18, 12, 14, 14, 35, 14, and 5 protein sequences from *Glycine max*, *Brassica napus*, *Arabidopsis thaliana*, *Oryza sativa*, *Zea mays*, *Triticum aestivum*, *Sorghum bicolor*, and *Solanum tuberosum*, respectively (Table S1). These sequences were used for protein alignments and the generation of a phylogenetic tree (Figure 2A). It can be seen that a small group of PLDs is separated from the others. This group named ζ consists of PLDs that possess a ‘Phox homology’ (PX) domain and a ‘pleckstrin homology’ (PH) domain at their N-terminal end; therefore, these PLDs are also named PX-PH-PLDs [42]. In the schematic representation of PLD primary sequences (Figure 2B), and in the modeled PLD structures obtained by AlphaFold2 (Figure 2C), the PX and PH domains are shown in green and brown, respectively. The other PLDs lacking such domains have an N-terminal-located C2 domain that is represented in purple in Figure 2B,C. Due to this difference in domain composition, PX-PH-PLDs and C2-PLDs differ in length. Considering the PLDs represented in Table S1, the mean molecular weights of PX-PH-PLDs and C2-PLDs are 120 kDa and 95 kDa, respectively. C2-PLDs can be sub-clustered into α-, β/γ-, ε-, and δ-subtypes (Figure 2). 

**Figure 3:**
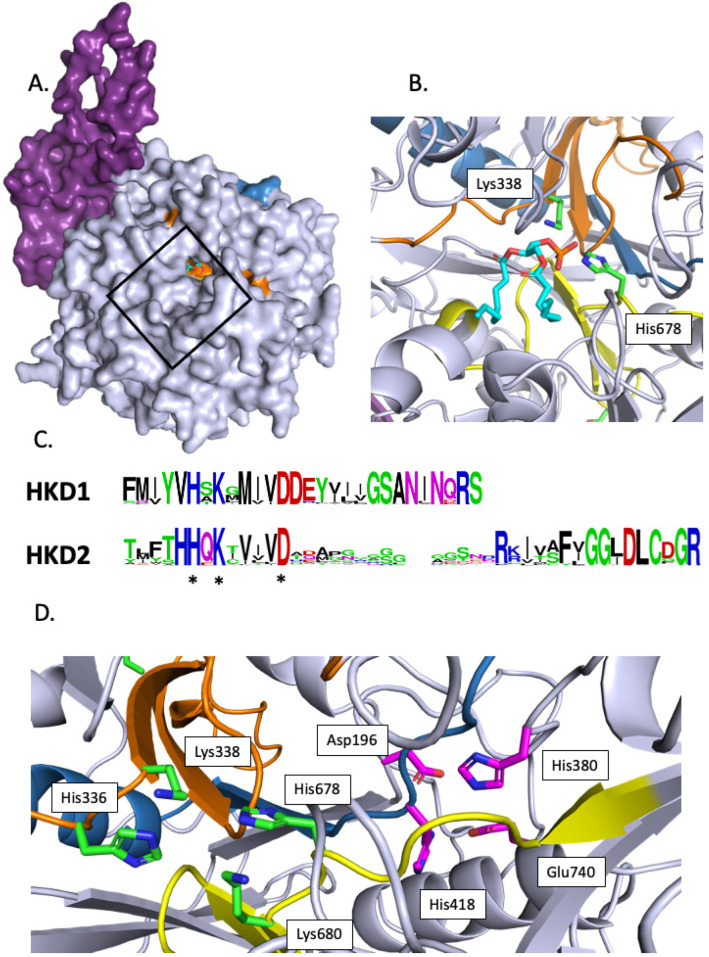
Structural features of the active site of selected plant PLDs: (**A**) Predicted structure of TaPLD1. The black rectangle indicates the substrate binding pocket. The molecule inside the pocket is a diC8-PA. We positioned it by overlapping the predicted TaPLD1 sequence with that of AtPLDα1 crystallized with diC8-PA [43]. (**B**) Detailed view of diC8-PA inside the active site. (**C**) Consensus motifs of HKD1 and HKD2. HKD1 and HKD2 refer to the first and second HKD domain starting from the N-terminus part of PLD. Asterisks (*) indicate the conserved His, Lys, and Asp. (**D**) Detailed view from the active site of TaPLD1. Colored in green are the lateral chains of the catalytic His, Lys, and Asp residues of the HKD domains shown within the TaPLD1 structure. Colored in magenta are the lateral chains of the residues involved in calcium binding as shown in [43]. Purple, C2 domain; orange, first HKD domain; yellow, second HKD domain; red, PI-4,5-P2 binding region 1 (PBR1 domain) [44].

In Arabidopsis, the crystallization of PLDα1 with diC8-PA allowed the identification of the substrate binding pocket [43]. This pocket is found in all PLDs, and it is open to the exterior (Figure 3A, illustrating this pocket in TaPLD1). 

All PLDs possess two copies of the domain HxKxxxxD denoted ‘HKD’ from the conserved His, Lys, and Asp residues. The HKD1 and HKD2 consensus motifs of the PLDs in Table S1 were calculated and are represented in Figure 3B. In the first HKD domain, the His, Lys, and Asp residues are conserved in 97.7%, 99.2%, and 99.2% of the sequences of Table S1; this calculation did not take into account ZmPLD6 for which no HKD1 domain aligned with the other sequences. In the HKD2 domain, the His, Lys, and Asp residues are conserved in 99.2%, 98.4%, and 100% of the sequences of Table S1; this calculation did not take into account BnaPLDa1A5 and TaPLD27 for which no HKD2 domain aligned with the other sequences. In the predicted structure of TaPLD1 (Figure 2C and Figure 3C), one can see that even though separated by 297 residues in the primary amino acid sequence, the two HKD domains are close in the 3D structure, with His and Lys of one motif facing the Lys and His of the second motif (Figure 3C). These residues are within the substrate binding pocket with the two HKD motifs associating to produce a single active site. 

C2-PLDs possess a C2 domain composed of an eight-stranded beta sandwich constructed around a conserved four-stranded motif. The consensus motifs for the C2 domains of α and non-α-subtype C2-PLDs (see Table S1) were identified (Figure S2A). These C2 domains were found to differ by the presence of Asp residues at specific positions in non-α-subtype C2-PLDs but missing in α-subtype C2-PLDs. This leads to Ca^2+^ binding loops that differ in their electrostatic properties (Figure S2B,C).

**Figure 4:**
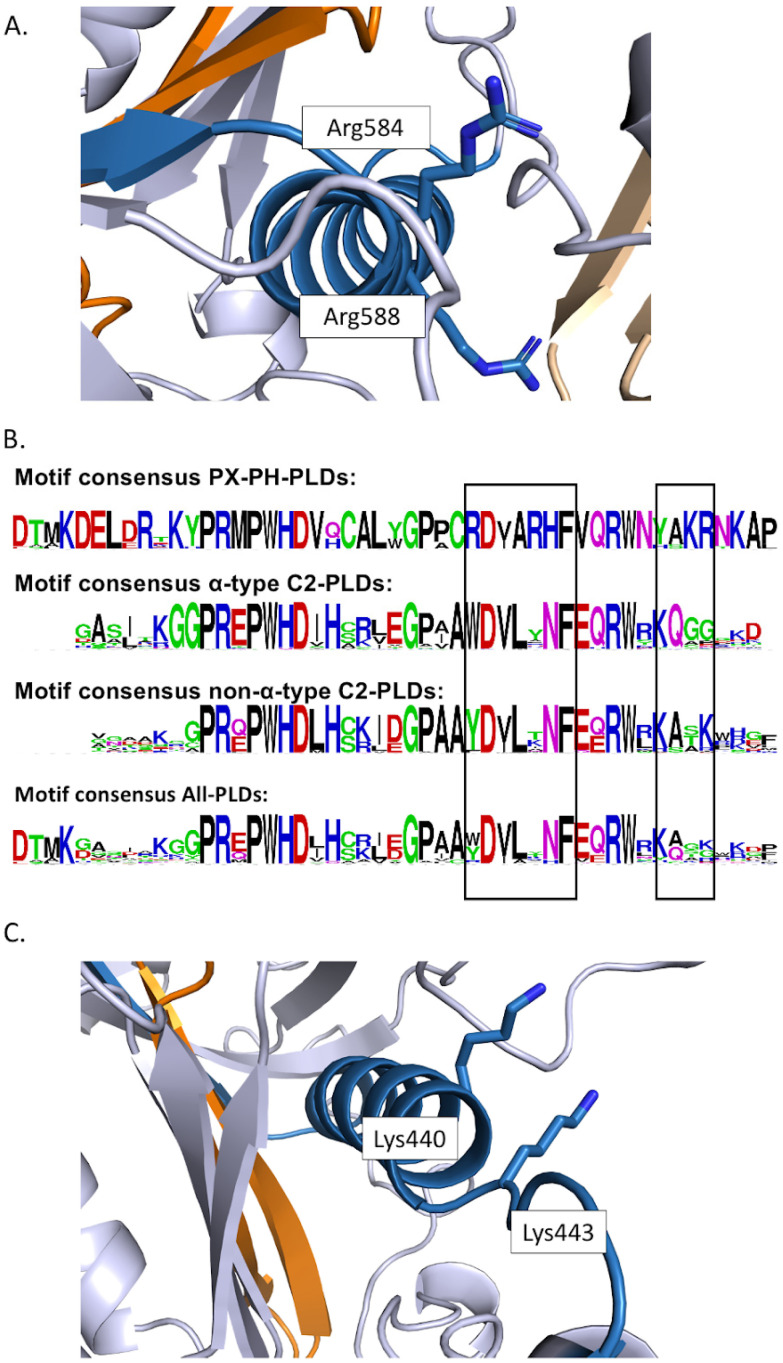
Structural features of the PBR1 domain in plant PLDs: (**A**) Positions of the Arg in the ARxARFH submotif of the PBR1 domain of PX-PH-PLDs. The lateral chain of the Arg of the ARxARFH submotif of TaPLD9 is colored blue. The PBR1 domain is blue. (**B**) Motif consensus of PBR1. Consensus was calculated considering all PLDs of Table S1, but also considering only PX-PH-PLDs, α-subtype C2-PLDs, and non-α-subtype C2-PLDs. (**C**) Positions of the Lys in the PBR1 domain of TaPLD10, a non-α-subtype C2-PLDs.

Sciorra et al. [45] identified a highly conserved motif located between the two HKD domains. This domain was named PI-4,5-P_2_ binding region 1 (PBR1) and is enriched in basic amino acids [45]. In this region, two Arg residues (corresponding to Arg554 and Arg558 of mouse PLD2 [45]) situated in a **R**DxA**R**HF submotif were shown to be necessary for enzyme activation by PI-4,5-P_2_. In the predicted PX-PH-PLD (TaPLD9) structural model, the RDxARHF submotif is part of a helix (Figure 4A). We aligned the PBR1 motifs of plant PLDs retrieved for this article and drew the consensus motif for different classes of plant PLDs (Figure 4B). Interestingly, the RDxARHF submotif was present in the PX-PH-PLDs of Table S1. Except for the Asp, these residues were not conserved in C2-PLDs. 

Interestingly, non-α-subtype C2-PLDs possess another motif, the KxxK submotif, located at the C-terminal side of the PBR1 region (Figure 4B,C). In non-α-subtype C2-PLDs, the first Lys was found in 88% of the concerned PLDs within our list (or replaced by an Arg in 8% of them), while the second Lys was found in 85% of the concerned PLDs (and replaced by an Arg in 10% of them); for α-subtype C2-PLDs, the first Lys was found in 90% of the concerned PLDs within our list (and replaced by an Arg in 5% of them), but the second Lys was never present. 

In the crystallized Arabidopsis AtPLDα1, a Ca^2+^ ion was found in the active site, coordinated by four residues (Asp187, His372, His 406, and Glu722). These residues were conserved in the primary sequence of all of the C2-PLDs of Table S1 (Figure S3A). If we consider, for example, the structure of TaPLD1, an α-subtype C2-PLD, and we overlap it with that of crystallized AtPLDα1 [43], the 4 Ca^2+^-coordinating residues overlap with those of AtPLDα1. These four residues were also found aligned in the primary amino acid sequence of the PX-PH-PLDs (Figure S3A), and their positions in the active site of the predicted TaPLD1 structure are shown (Figure 3C and Figure S3B).

#### 2.1.2. Diacylglycerol Kinases

Genes encoding DGKs have been found in the genomes of many plant species, including *Oryza sativa* [46], *Zea mays* [47], *Brassica napus* [48], and *Glycine max* [49]. We retrieved 7, 15, 10, 6, 15, 23, 8, and 2 protein sequences from *Arabidopsis thaliana*, *Brassica napus*, *Glycine max*, *Oryza sativa*, *Zea mays*, *Triticum aestivum*, *Sorghum bicolor*, and *Solanum tuberosum*, respectively (Table S2).

**Figure 5:**
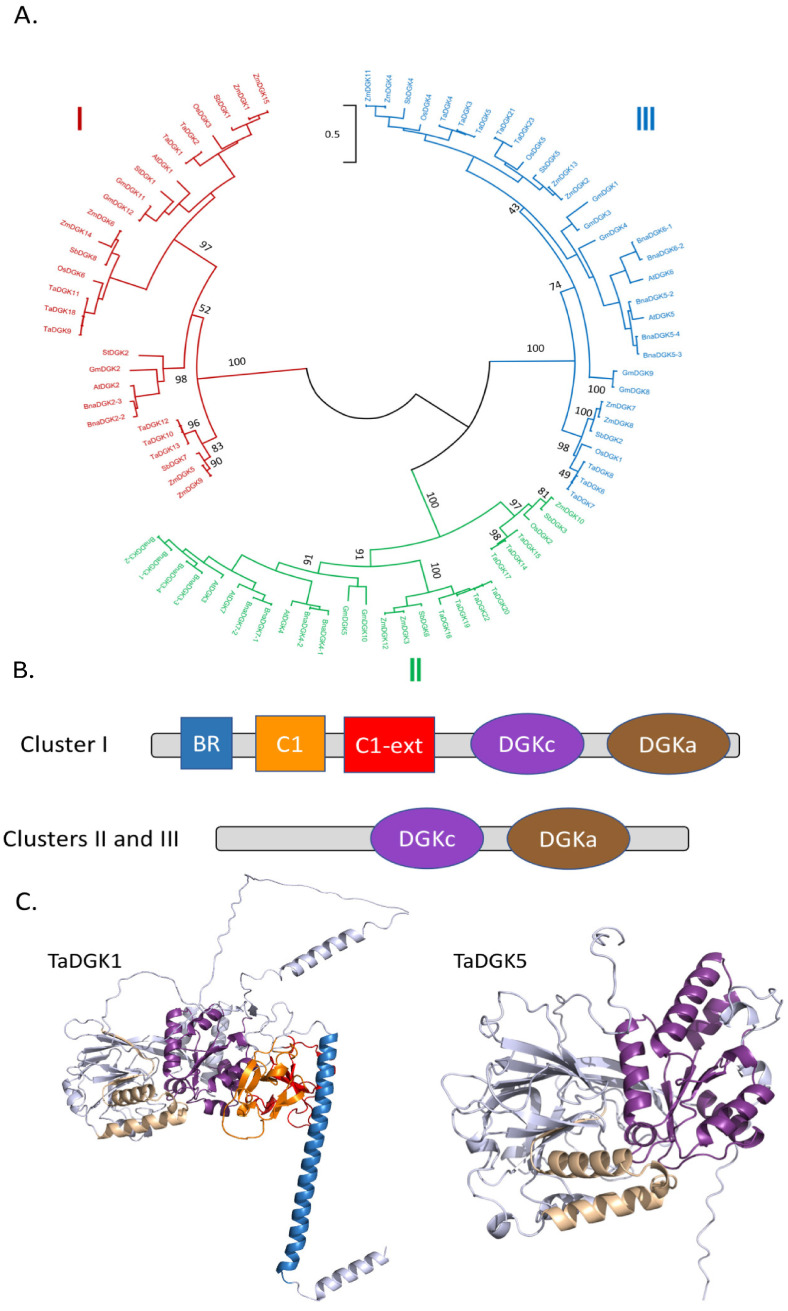
Structural features of plant DGK family members: (**A**) Phylogenetic tree showing different DGKs. The scale bar refers to a phylogenetic distance that is the average number of substitutions per site (here 0.5). Numbers on the branches indicate bootstrap percentage after 1000 replications in constructing the tree. (**B**) Schematic representation of the structural domains in plant DGKs. Purple, DGKc; brown, DGKa; blue, N-terminal basic region; orange, C1 domain; red, C1 extended domain. (**C**) Structural differences between cluster I-DGKs and cluster II/III-DGKs. TaDGK1, was chosen as a representative of a cluster I DGK, while TaDGK5 was chosen as a representative of a cluster II/III DGK. Purple, DGKc; brown, DGKa; blue, N-terminal basic region; orange, C1 domain; red, C1 extended domain. The Alphafold2 structures can also be colored based on the confidence with which each domain is modeled (Figure S5).

Sequences were aligned and used to generate a phylogenetic tree. DGKs were clustered into three groups (Figure 5A). Cluster I DGKs possess an N-terminal basic region (BR) represented in blue in Figure 5B,C. Cluster I DGKs also possess two C1 domains, the most C-terminal one being extended (longer) (Figure 5B). C1 domains are 50-residue modules containing two small β-sheets and a short C-terminal helix (Figure 5C).

**Figure 6:**
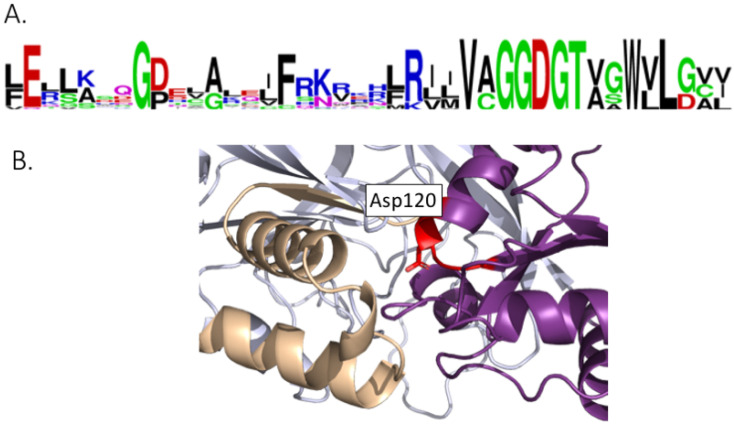
Structural features of the GGDG motif of the active site of DGKs: (**A**) Motif conservation of the GGDG region. (**B**) Structure of the GGDG region of TaDGK5. The GGDG region appears in red. It is located within the DGKc domain (in purple); it is at the interphase with the DGKa domain (in brown).

All DGKs possess a DGK-catalytic (DGKc) domain and a DGK-accessory (DGKa) domain and together they make up the catalytic domain [50]. As expected, these domains are situated close together in the predicted structure (Figure 5C). A GGDG motif (with the characteristics for ATP binding) is required for DGK catalysis (Figure 6A), and it is conserved in all DGK sequences given in Table S2 except for GmDGK1 and OsDGK3. This motif is located in the DGKc domain, at the interface with DGKa (Figure 6B).

Cluster II and III DGKs share the same basic domain organization, with DGKc and DGKa domains within the C-terminal part of the proteins. Yet, some cluster III DGKs contain a C-terminal calmodulin binding domain (CBD) due to alternative splicing [51].

Cluster I DGKs are longer than ClusterII/III DGKs because of differences in domain composition and the presence of a non-structured regions. Considering the selected DGKs in Table S2, the mean molecular masses of DGKI and DGKII/III are 78 kDa and 55 kDa, respectively.

#### 2.1.3. PI-PLCs

Phosphoinositide-specific phospholipases C (PI-PLCs) cleave, in a Ca^2+^-dependent manner, membrane PI-4,5-P_2_ to produce two second messengers: (i) a lipid, DAG, and (ii) a polar molecule, inositol 1,4,5-triphosphate (IP_3_). In plants, it has been suggested that not only PI-4,5-P_2_ but also phosphatidylinositol-4-phosphate (PI-4-P) could be substrates of PI-PCs [52]. In animals, the role and regulation of PI-PLC isozymes are well established [53]. The canonical model states that PI-PLC action is linked to the activation of protein kinase C (PKC) by DAG and to an intracellular Ca^2+^ release mediated by IP_3_-sensitive channels (IP_3_ receptors). The mode of action of plant PI-PLCs must be different from the animal model since the amount of PI-4,5-P_2_ is lower in plant membranes [54], plants apparently lack conventional IP_3_ receptors, and no direct plant PKC orthologs have been identified to date. Most likely the role of PI-PLC in plants is mediated by the phosphorylation of DAG to PA, and via phosphorylation of IP_3_ to highly phosphorylated forms such as inositol hexaphosphate IP_6_ [20]. 

Genes encoding PI-PLCs have been found in the genomes of many crop species, including *Oryza sativa* [55], *Zea mays* [56], *Brassica napus* [57], and *Glycine max* [58]. We retrieved 22, 9, 13, 5, 5, 7, 6, and 1 protein sequences from *Brassica napus*, *Arabidopsis thaliana*, *Glycine max*, *Oryza sativa*, *Zea mays*, *Triticum aestivum*, *Sorghum bicolor*, and *Solanum tuberosum*, respectively (Table S3).

**Figure 7:**
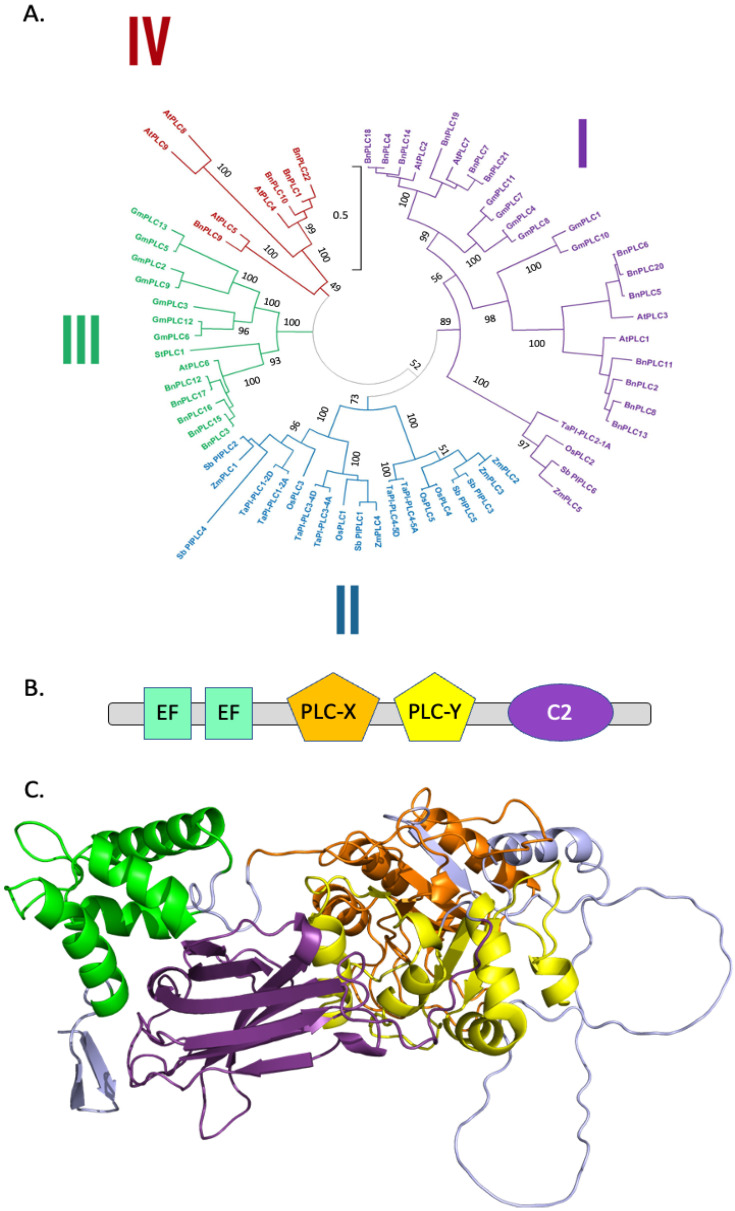
Structural features of plant PI-PLC family members: (**A**) Phylogenetic tree of different PI-PLCs. The accession numbers of the protein sequences used can be found in Table S3. The scale bar refers to a phylogenetic distance that is the average number of substitutions per site (here 0.5). Numbers on the branches indicate bootstrap percentage after 1000 replications in constructing the tree. (**B**) Schematic representation of the structural domains in plant PI-PLCs. Purple, C2 domain; green, PX domain; green, EF domain; orange, PLC-X domain; yellow, PLC-Y domain. (**C**) Structural model of TaPI-PLC2-1A. Purple, C2 domain; green, PX domain; green, EF domain; orange, PLC-X domain; yellow, PLC-Y domain. The Alphafold2 structures can also be colored based on the confidence with which each domain is modeled (Figure S5).

The PI-PLC sequences were aligned, and a phylogenetic tree was generated (Figure 7A). PI-PLC sequences were apparently clustered into four groups. Yet, what is striking in the tree is the low sequence diversity as indicated by the bar (which reflects phylogenetic distance). 

All plant PI-PLCs have the following structural composition; two EF hands, a PLC-X and a PLC-Y domain, and a C2 domain (Figure 7B). The structural model of TaPI-PLC2-1A is displayed (Figure 7C).

**Figure 8:**
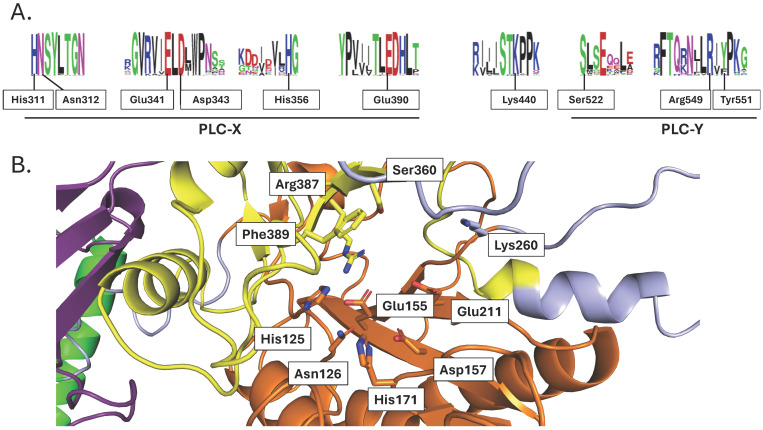
Structural features of the catalytic region of crop PI-PLCs: (**A**) Motif conservation of catalytic residues. Numbering refers to the residues of rat PLCδ1. (**B**) Structure of the region containing the catalytic residues of TaPI-PLC-2-1A.

Two regions homologous across all PI-PLCs, either eukaryotic or prokaryotic ones, are denoted as PLC-X and PLC-Y domains. They have been shown to be important for the catalytic activity of PI-PLCs. In rat PLCδ1, key amino acids for catalysis have been identified (His311, Glu341, Asp343, His356, Glu390, Lys440, Ser522, Arg549, and Tyr551) [59]. These residues are well conserved amongst the crop PI-PLCs of Table S3 (Figure 8A) and belong to predicted PLC-X and PLC-Y regions. These two regions face each other in the 3D structure, and the identified residues for catalysis are at their interface plane (Figure 8B). 

The C2 domain has the possibility to coordinate Ca^2+^, and Ca^2+^ has been seen in the C2 domain of crystallized rat PLCδ1 [60] and its Ca^2+^ binding loop is rich in Asp (see discussion above about C2 in PLDs). This is also the case for crop plant PI-PLCs, as seen in the conservation motif (Figure S4A) and exemplified by TaPI-PLC2_1A (Figure S4B). Furthermore, in the crystallized rat PLCδ1, a second Ca^2+^ ion was detected in the active site and coordinated by Asn312, Glu341, Asp343, and Glu390 (rat PLCδ1) [60]. Interestingly, these residues are conserved in crop species PI-PLCs (Figure 8A); they correspond to Asn126, Glu155, Asp157, and Glu211 in TaPI-PLC2-1A, respectively (Figure 8B). Their role in Ca^2+^ binding is yet to be established.

In conclusion, PI-PLCs, DGKs from all three clusters, and PLDs from both classes, C2-PLDs (comprising α- β/γ-, ζ- and δ- subclasses) and PX-PH-PLDs, are clearly present in crop plants. These enzymes show conserved key structural features that determine catalytic properties/regulation as seen in homologous proteins of model plants. Therefore, signaling processes relying on these enzymes are likely to play similar roles in crop plant physiology and acclimation to environmental stresses.

### 2.2. PA Binding Proteins—A Focus on NADPH Oxidase

PA primarily acts as a regulator of plant physiology via its binding to specific proteins associated with different cell functions. To date, the best documented PA-binding proteins in plants are the NADPH oxidases belonging to the respiratory burst oxidase homolog (RBOH) family. In Arabidopsis, PA binds to RBOHD (At5g47910) [17], and this stimulates in vitro RBOHD activity. 

**Figure 9:**
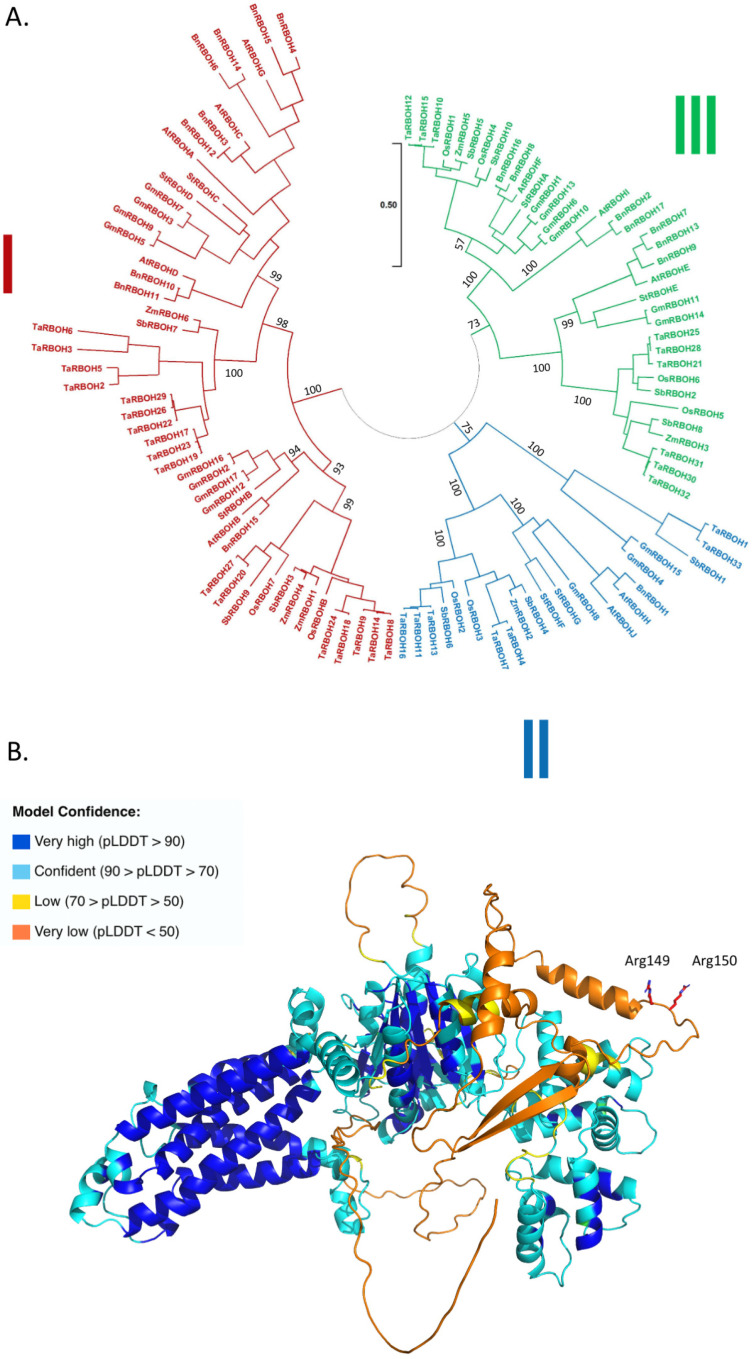
Structural features of RBOH orthologs: (**A**) Phylogenetic tree of different RBOHs. The accession numbers of the protein sequences used can be found in Table S4. The scale bar refers to a phylogenetic distance that is the average number of substitutions per site (here 0.5). Numbers on the branches indicate bootstrap percentage after 1000 replications in constructing the tree. (**B**) Predicted structure of Arabidopsis AtRBOHD. The structure was predicted by AlphaFold2. The color code corresponds to model confidence as defined by AlphaFold2. AlphaFold2 produces a per-residue confidence score, named “predicted local distance difference test” (pLDDT), which ranges between 0 and 100. The Alphafold2 structures can also be colored based on the confidence with which each domain is modeled (Figure S5).

In the scope of the article, we wanted to illustrate that crop species, like model plants, contain proteins that bind PA and for this, we focused on RBOH. We retrieved 17, 10, 17, 8, 6, 33, 10, and 7 protein sequences from *Brassica napus*, *Arabidopsis thaliana*, *Glycine max*, *Oryza sativa*, *Zea mays*, *Triticum aestivum*, *Sorghum bicolor*, and *Solanum tuberosum*, respectively (Table S4). The RBOH sequences were aligned, and a phylogenetic tree was generated (Figure 9A). RBOH sequences were clustered into three groups.

In *Arabidopsis thaliana*, Arg149 and Arg150 of AtRBOHD have been identified as important for PA binding. They are lacking in AtRBOHF, and when introduced, the binding of PA to this protein was greatly enhanced [17].

AtRBOHD structure was predicted by AlphaFold2 (Figure 9B). Arg149 and Arg150 appear to be on the surface of the protein, located in a part for which the model confidence is very low. The alignment of primary sequences only gives a conservation of this Arg doublet for BnRBOH10 and BnRBOH11.

Based on sequence identity and structural modeling, it appears probable that PA binding to RBOHD NADPH oxidase is conserved in both model and crop plants. That said, biochemical data should be obtained to help confirm this conclusion.

## 3. Discussion

From our study and others, there is no doubt that lipid signaling pathways are present in crop plants. Common protein targets of PA also exist in crop plants. It is expected that the physical and intrinsic properties of PA will influence the state of membranes and favor membrane curvature and the formation of vesicles [61] whatever the plant species. The question remains as to whether PA and lipid signaling in crop species fully mirror the currently studied roles in Arabidopsis and other model plants. Understanding the mode of regulation of enzymes implicated in PA production will require the in-depth deciphering of their structural features. 

### 3.1. Structural Features of PLDs

From the present article, we can see that crop PLDs can be either C2-PLDs or PX-PH-PLDs. It should be noted that mammalian cells have no C2-PLDs [62]. It can also be seen that monocot and dicot PLD sequences are present in all C2 subtypes, thus suggesting that class diversification occurred early in evolution, before the separation of monocot from dicot species. Concerning ε-PLDs, they share a common ancestor with α-PLDs, but they may have diverged before the monocot–dicot separation. In Arabidopsis, some PLDs have been named γ-PLDs [63]. Our phylogenetic tree places γ-PLD sequences with dicot β-PLDs, whereas there is a clear separation of β/γ-PLDs from dicot species and β-PLDs from monocot species. This suggests that γ-PLDs appeared after the monocot–dicot separation and γ-PLDs are actually a subclass of β-PLDs in dicot species.

PLDs have different catalytic properties based on their requirement for Ca^2+^ or their activation by phosphatidylinositol-4,5-bisphosphate (PI-4,5-P_2_). The α-subtype PLDs require high Ca^2+^ concentrations (in the mM range) while they are insensitive to PI-4,5-P_2_. On the other hand, β/γ-subtype PLDs are activated by low Ca^2+^ concentrations (in the μM range) and by PI-4,5-P_2_ [64]. Interestingly, PLDδs are not only activated by Ca^2+^ and PI-4,5-P_2_ but also by an unsaturated fatty acid (oleic acid) [65]. Finally, PLDζs are PI-4,5-P_2_-dependent and Ca^2+^-independent [64]. Such properties might be explained by the domains that constitute the different PLD subtypes. Yet, this current article shows that some questions remain unanswered. 

For instance, what is the exact role of the C2 domain in the binding of Ca^2+^ in C2-PLDs? In the known C2 domain, a disordered loop of *circa* 22 residues connecting β1 and β2 makes up the Ca^2+^-binding site [66]. The C2 domain is believed to be involved in Ca^2+^-dependent phospholipid binding, as a membrane tethering mechanism [66]. That said, no Ca^2+^ binding was found in the C2 domain structure of crystallized Arabidopsis PLDα1, even though 200 mM Ca^2+^ was present during crystallization [43]. The C2 domains from the C2-PLDs of our list were found to differ by the presence of Asp residues at specific positions in non-α-subtype C2-PLDs but were missing in α-subtype C2-PLDs. This leads to Ca^2+^ binding loops that differ in their electrostatic properties. That might result in α-subtype C2-PLDs having a C2 domain that does not bind Ca^2+^ or binds it with a lower efficiency than that of non-α-subtype C2-PLDs. Yet, whether or not Ca^2+^ actually binds to the C2 domain even in non-α-subtype PLDs still requires more investigation, such as crystallization studies and in silico modeling. It has been proposed that when cytosolic Ca^2+^ is low (resting state), β-subtype PLD is anchored to the membrane because of specific interactions between its N-terminal C2 domain and PI-4,5-P_2_. When Ca^2+^ increases (for instance in response to stress), this leads to Ca^2+^ binding to the C2 domain of β-subtype PLD, resulting in conformational changes [67]. As a consequence, PI-4,5-P_2_ binding to the C2 domain becomes weakened, whereas binding to the PBR1 would be strengthened. This conformational change would also enhance the binding of PC (a substrate of PLDs) to the C2 domain. Indeed, an analysis of the Arabidopsis PLDα1 crystal structure revealed that C2 produces a surface interaction with the HKD2 subdomain (defined in [43]) corresponding to residues 460–725 of AtPLDα1, mainly via hydrophobic residues. Mutating these residues impaired PLDα1 activity and suggested that the C2–HKD2 interaction is essential for AtPLDα1 enzymatic activity. Hence, the C2 domain might regulate the conformation of the substrate binding pocket, controlling substrate entry and/or product release [43].

In Arabidopsis AtPLDα1, mutation of any one of the four residues that coordinate a Ca^2+^ ion in the active site (Asp187, His372, His 406, and Glu722) led to the complete loss of catalytic activity [43]. The Ca^2+^ appeared to be involved in an interaction network impacting the positions of the catalytic site and substrate-binding pocket residues [43]. These residues were conserved in the primary sequence of all of the C2-PLDs of Table S1 (Figure S3A). These four residues were also found aligned in the primary amino acid sequence of the PX-PH-PLDs. Yet, PX-PH-PLDs are Ca^2+^-independent [64] and non-α-subtype C2-PLDs require less Ca^2+^ than α-subtype C2-PLDs [63]. Therefore, more studies are still necessary to understand the structural determinants of the link between Ca^2+^ and PLDs.

In plants, the activation of PLD by the binding of phosphoinositides is not yet fully elucidated and many questions await answers. What is the role of the PX domain in plant PX-PH-PLDs? As already mentioned, PX refers to a ‘Phox homology’ domain (PX domain) in PX-PH-PLD proteins. This domain consists of approximately 120 residues, and it is composed of three antiparallel β-strands followed by three alpha helices [62]. It is a phosphoinositide binding module found in many human proteins with diverse functions in cell signaling, vesicular trafficking, protein sorting, and lipid modification [68]. The exact role of the PX domain of PLDs in plant cells requires further attention. Deletion of the PX region led to a mislocalization of human PLD1, but it did not prevent PLD catalytic activity in vivo [69]. 

The ‘pleckstrin homology’ (PH) domain is made up of about 100 residues consisting of two perpendicular anti-parallel β sheets, followed by a C-terminal amphipathic helix. The PH domain is also believed to be involved in binding phosphoinositides and similar to the PX domain, it is found in a wide range of proteins associated with intracellular signaling or as constituents of the cytoskeleton in mammals [70]. PH domains might be involved in the targeting of PH-containing proteins to PI-4,5-P_2_-enriched membranes. Indeed, some mammalian and yeast PLDs appear to interact with PI-4,5-P_2_ via the PH domain [71]. In plants PI-4,5-P_2_ is mostly present in plasma membrane microdomains [23,72]. However, Sciorra et al. [45] showed that the N-terminal PH domain was not involved in the activation of mammal PLD1 and PLD2 by PI-4,5-P_2_. The role of the PH domain of PLDs might not be common to different PLDs, and more studies are required to assess the role of this domain in the context of plant PLDs. 

Other structural features of PLDs might be responsible for their regulation by PI-4,5-P_2_, such as the PBR1 domain. In this region, two Arg residues (corresponding to Arg554 and Arg558 of mouse PLD2 [45]) situated in an RDxARHF submotif were shown to be necessary for enzyme activation by PI-4,5-P_2_. Due to the conservation of the two Arg residues in PX-PH-PLD, it is highly probable that they also explain the activation by PI-4,5-P_2_ for this subclass. This appears to agree with the critical role of Arg and Lys for phosphoinositide binding [73]. It nevertheless needs to be experimentally proven. The RDxARHF submotif of PBR1 was not conserved in the non-α-subtype C2-PLDs that possess another submotif, the KxxK submotif, located at the C-terminal side of the PBR1 region. Zheng et al. [44] showed that these two Lys residues were necessary for the activation by PI-4,5-P_2_ of AtPLDβ enzymatic activity. Can it be generalized that the KxxK submotif of PBR1 is involved in the binding of phosphoinositide to non-α-subtype C2-PLDs?

Finally, it is worth noting that neither the RDxARHF submotif nor the KxxK submotif was conserved in α-subtype C2-PLDs, which are not activated by PI-4,5-P_2_.

### 3.2. DGKs

In the phylogenetic tree, DGKs were clustered into three groups, with monocot and dicot sequences present in each, thus suggesting that the different DGK forms had appeared before the separation of monocot and dicot species.

Concerning the N-terminal basic region (BR) present in Cluster I DGKs, this region is predicted to be a transmembrane helix [74]. We have shown that the N-terminal basic region of Arabidopsis DGK1 and DGK2 was sufficient to address fusion proteins to endoplasmic reticulum (ER) membranes [74]. The role of the two C1 domains present in plant Cluster I DGKs is not clear. The C1 domain of mammal protein kinase C (PKC) binds DAG. However, the absence of C1 domains in cluster II and III DGKs does not impede their catalytic activity, and therefore its role does not appear related to binding DAG as a substrate [75]. Therefore, the role of C1 domains in DGKs is proposed to be linked to the fine targeting of DGKs to membrane domains, and not catalysis per se. Indeed, both C1-domains of cluster I DGKs are structured next to the predicted transmembrane BR region and are thus likely to interact with membranes.

### 3.3. PI-PLCs

For PI-PLCs, the phylogenetic tree differed from those obtained with either PLD or DGK sequences. Comparing the distance scales, it appears that PLC sequences are less diversified than PLD or DGK sequences. All plant PI-PLCs have the following structural composition; two EF hands, a PLC-X and a PLC-Y domain, and a C2 domain. In that way, plant PI-PLCs are structurally similar to mammalian PLC isoforms ζ (which consist of these domains only, in this order). 

Concerning the residues involved in the catalysis of PI-PLC (Figure 8), some discrepancies occur within the conserved residues. *Sorghum bicolor* SbPI-PLC4 contains an Ala, Trp, and Asn instead of the equivalent Asp343, Arg549, and Tyr511 of rat PLCδ1, respectively. In Arabidopsis, AtPLC8 and AtPLC9 have Leu, Pro, Lys, Arg, Gly, and Arg instead of the equivalent His311, His356, Glu390, Lys440, Ser522, and Tyr511 of rat PLCδ1, respectively. Mutations of His311 and His356 in rat PLCδ1 resulted in a dramatic reduction in PIP_2_ binding [59]. Yet, there is no experimental evidence showing that Arabidopsis PLC8 or PLC9 is catalytically inactive. On the contrary, the role of AtPLC9 in heat stress resistance has been demonstrated using mutant studies in Arabidopsis [76]. Similarly, the overexpression of AtPLC9 increased drought tolerance in Arabidopsis [77]. One can assume that the catalytic mechanism of these enzymes is different or that they bear functionality independent of PLC activity (i.e., due to “moonlighting”). 

The EF-hand domains of PI-PLC consist of a twelve-residue loop flanked on both sides by a twelve residue α-helical domain. In an EF-hand loop, Ca^2+^ is coordinated in a pentagonal bipyramidal configuration. The basic functional unit of EF-hand proteins is usually a pair of EF-hand motifs that form a stable four-helix bundle domain. The activity of PI-PLCs is Ca^2+^-dependent [20]. Since these enzymes contain EF-hand domains, it was presumed they were responsible for the Ca^2+^ sensitivity of PI-PLC. Yet, rat PLCδ1 crystallized with Ca^2+^ did not show Ca^2+^ associated with the EF hands [60]. Consistently, the residues important for Ca^2+^ binding to the loop of the EF hands were missing in the EF hands of PI-PLCs, including those from crops. Therefore, the role of the EF-hand domain is still not established. N-terminally truncated AtPLC2 lacking a part of an EF-hand domain was inactive, while retaining the ability to bind PI-4,5-P_2_-containing vesicles The role of the EF-hand domains could be to assist in the formation of the PI-PLC active site, as in animal PI-PLCs [78]. Note that the C2 domain, while being C-terminal and separated from the N-terminal EF hands in the primary sequence, is actually close to the EF hands in the 3D structure. 

Ca^2+^ has been seen in the C2 domain of crystallized rat PLCδ1 [60]. Its Ca^2+^ binding loop is rich in Asp (see discussion above about C2 in PLDs). This is also the case for crop plants (Figure S4). This might be a site for calcium binding. Concerning the residues involved in coordinating the Ca^2+^ ion detected in the active site of the crystallized rat PLCδ1, they are conserved in crop species PI-PLCs. Their role in Ca^2+^ binding is yet to be experimentally established.

In conclusion, does Ca^2+^ bind to both the C2 domain and the active site as shown for rat PLC, or is this just a crystallization artifact? What happens if we mutate residues involved in Ca^2+^ binding to C2 domains? Which mode of binding can explain the strict Ca^2+^ dependency of PI-PLCs? Structural studies combining X-ray analysis of crystallized proteins and simulations of molecular dynamics should help answer these questions.

### 3.4. PA Binding Proteins

A list of PA-binding proteins has been published recently [31]. PA has been shown to bind to protein kinases, including MPKKs (mitogen-activated protein kinase kinases) (AtMKK7 (At1g18350), AtMKK9 (At1g73500), and MPKs (mitogen-activated protein kinases) (AtMPK3 (At3g45640)/AtMPK6 (At2g43790). The roles of these MPKKs and MPKs are well established in Arabidopsis responses to salt stress [79] and hypoxia [80]. PA also binds to another protein kinase called AtPINOID (At2g34650) involved in the control of PIN2 auxin-transporter activity in response to salt stress [81]. PA can also bind to lipid kinases such as AtSPHK1 (At4g21540) and AtSPHK2 (At4g21534), which are sphingosine kinases. These enzymes could potentially be involved in the production of phytosphingosine-1-phosphate acting in ABA-mediated stomata closure [82]. In Arabidopsis, PA also binds to several more protein kinases linked to salt tolerance including a sucrose non-fermenting-1-related protein kinase 2.4 AtSnRK2.4 (At1g10940) [83] and Salt Overly Sensitive 2 SOS2 [36]. PA binding promotes kinase activity and plasma membrane localization of SOS2, thus activating the SOS1 plasma membrane Na^+^/H^+^ exchanger to allow Na^+^ efflux from the cells to relieve Na^+^ toxicity [36]. PA can also bind transcription factors; the binding of PA to a werewolf (WER), an R2R3 MYB transcription factor, in Arabidopsis, affects its nuclear localization [84]. PA can also bind to ion channels, such as the potassium channel OsAKT2 (Os05g35410) in rice [85]. PA can also bind to hormone receptors such as OsGID1 (Os05g33730), a gibberellin receptor [86]. Arabidopsis Arginase 2 has a potential role in both polyamine and proline biosynthesis; identified as a PA binding protein, in vitro arginase activity was enhanced by PA binding [87]. The list of PA protein targets is constantly being updated suggesting that the complexity of PA signaling networks in plants has not been fully determined and understood. Some of these PA-binding proteins have been identified in crop species, such as rice, while others remain to be identified.

A PA-RBOHD interaction has also been linked to ABA-induced ROS production since Arabidopsis mutants either lacking PLDα1 or expressing non-PA-binding RBOHD were both compromised in ABA-mediated ROS production [17]. PA other than the PLD-derived one could be implicated in RBOHD binding. Recently, the role of DGK5 in producing a PA that stabilizes and activates RBOHD leading to a ROS burst in response to immune stimulation was reported in Arabidopsis [18]. Therefore, activation of RBOH activity appears to be a major transducing process of PA.

We retrieved different RBOH sequences from our species of interest. RBOH sequences are clustered into three clusters, I II, and III. Each cluster is divided into two subclusters. All subclusters contain sequences from monocots and dicots. AtRBOHA, AtRBOHC, AtRBOHD, and AtRBOHG are in one subcluster of cluster I, and AtRBOHB in the other; AtRBOHH and AtRBOHJ are in one subcluster of cluster II; AtRBOHF and AtRBOHI are in one subcluster of cluster III, and AtRBOHE in the other. One subcluster of cluster II only comprised four sequences, from only three species (*Sorghum bicolor*, *Triticum aestivum*, and *Glycine max*). 

In *Arabidopsis thaliana*, AtRBOHD and AtRBOHF have been shown to bind PA. PA binding to RbohD was stronger than binding to RbohF [17]. No consensus sequence for the PA binding domain exists; but common characteristics exist, which are tightly grouped basic residues that in some proteins are placed adjacent to a hydrophobic stretch [14]. Arg149 and ArgA150 of AtRBOHD were shown to be important in the binding of PA to this protein [18]. In the predicted structure of AtRBOHD, these two Arg are at the surface of the protein; they belong to a protein structure for which the model confidence is very low (Figure S5). When we performed primary sequence alignments, only two proteins, very phylogenetically close proteins, BnRBOH10 and BnRBOH11, had an Arg doublet that aligned with that of AtRBOHD. Does that mean that RBOH from other species do not bind PA? It is unlikely. This doublet is not a prerequisite for PA binding since AtRBOHF lacks this Arg doublet but binds PA, even though less efficient than AtRBOHD. Moreover, we do not expect all RBOH orthologs to bind PA. Thus, it appears that primary sequence alignments or structural alignments are not enough to identify PA-binding RBOH. A thorough experimental work deciphering PA binding characteristics in the RBOH orthologs would be of high interest. Our structural characterization of the RBOH family indeed illustrates that understanding PA binding is the next frontier in the lipid signaling research field. It is a challenge because no PA domain consensus is described; thus, each protein family is likely to have its own PA binding motif; moreover, within one protein family, not all members are expected to bind PA. 

### 3.5. Involvement in Crop Responses to Environmental Stresses Responses

The presence of genes encoding enzymes involved in either lipid signaling or encoding PA target proteins is a clear indication that PA plays a role in crop plants. The differential expression of genes in response to environmental conditions could indicate when lipid signaling is involved. In *Glycine max* (soybean), genes encoding DGKs (*GmDGK1*, *GmDGK8*, *GmDGK9*, *GmDGK10*, *GmDGK11*, and *GmDGK12*) were induced within 24 h of a salinity treatment [49]. Transcriptomic profiling of soybean exposed to UV-B irradiation suggested that PA production via either *GmDGK5* or *GmDGK1* might be involved in high UV stress acclimation [88]. In *Zea mays*, all seven DGK-coding genes were induced following 12 h of cold stress exposure (4 °C). On the other hand, some maize DGKs were downregulated (*ZmDGK2*, *ZmDGK7*), whereas others (*ZmDGK1*, *ZmDGK3*, *ZmDGK4*) were induced in response to drought [47]. Interestingly, in response to a salt stress, known to involve a production of PA in Arabidopsis [79], most DGK-coding genes were downregulated in maize [47]. In contrast, a salt stress led to an upregulation of all six DGK genes identified in common bean plants (*Phaseolus vulgaris*) [89]. In *Brassica napus*, *BnDGK2-2*, *BnDGK2-3*, and *BnDGK3-3* were found to be induced by ABA and BR, while BR also induced *BnDGK7-1* and *BnDGK7-2* [48].

In *Glycine max*, several PI-PLC genes (*GmPLC6*, *GmPLC10*, and *GmPLC12*) were induced by ABA, and also by NaCl [58]. In *Brassica napus*, the expression of six PI-PLC genes were activated in response to dehydration, ABA, salt stress, or cold stress [57]. These changes, at least in part, could explain the observed increase in DAG content in leaves of *Brassica napus* following 24 h of dehydration or cold stress but not in response to ABA or NaCl [57]. Among the five PI-PLCs found in maize, the expression level of *ZmPI-PLC1* and *ZmPI-PLC1*3A increased following exposure to NaCl and osmotic stress [56]. *ZmPI-PLC3A* and *ZmPI-PLC3B* were also transiently upregulated in cold-treated maize plants. An overexpression of PI-PLC (homologous to ZmPI-PLC3A and 3B) led to the improved drought tolerance of maize [90].

In *Brassica napus*, the expression of *PLDδAnn* and *PLDδC7* was induced following exposure of plants to drought, NaCl, ABA, or cold (4 °C) [40]. In *Glycine max*, the expression of *GmPLDα1* and *GmPLDα2* was increased and that of *GmPLDγ* was decreased by salt stress [41]. 

It should be noted that gene expression changes do not necessarily mirror changes in enzyme activities. Instead, altered enzyme activities might rely on post-translational regulations, a mechanism that could involve Ca^2+^-dependent PLD activation [64]. That said, gene expression data are often well corroborated by mutant studies and biochemical assays that clearly demonstrate the role of lipid signaling enzymes in the stress acclimation of crop plants.

The overexpression of Arabidopsis PLDε (also named PLDα4) led to a stimulation of rapeseed (*Brassica napus*) [91] and soybean (*Glycine max*) [92] growth under nitrogen deficiency. This effect was attributed to the increased activity of enzymes involved in nitrate uptake and assimilation in PLDε-overexpressing lines. The mechanism leading to their activation by PLDε-derived PA remains unknown. GmPLDα1 may also play a role in nitrogen acquisition in soybean by promoting PA-dependent signal transduction implicating Nod factors leading to rhizobium–root interactions and subsequent root nodulation [93].

Interestingly, the knockdown of certain PLD genes has shown a positive effect on stress acclimation in crops. OsPLDβ1-knockdown rice plants were characterized by a basal accumulation of ROS and phytoalexins, suggesting that OsPLDβ1 was a negative regulator of rice stress responses. OsPLDβ1-knockdown rice plants exhibited an increased disease resistance to infections by *Pyricularia grisea* and *Xanthomonas oryzae* pv. *oryzae* pathogens [94]. In this case, it was not reported whether a basal activation of stress responses in OsPLDβ1-knockdown rice led to any growth penalty. Cumulative data suggest that PA production per se can by no means be correlated to an enhancement of plant stress resistance. Rather it is a balancing of PA production in different cell compartments by different enzymes giving rise to different molecular species of PA that are crucial to triggering plant adaptation responses [14]. 

Treatment with neomycin sulfate that interferes with PI-PLC activity via binding to phosphoinositides impaired the growth of maize seedlings due to a negative effect on photosynthesis and carbon metabolism pathways, as seen from a high-throughput RNA-seq analysis, diminished chlorophyll content, and net photosynthesis rates [95]. It has to be noted that neomycin sulfate might also affect PI-4,5-P2-dependent PLD [96] as well as other processes.

Besides acting in stress responses, PA is an important signaling molecule in plant growth regulation. In cotton, the binding of PA to HOX4 homeodomain-leucine zipper IV transcription factor interferes with its nuclear localization and prevents its role in regulating cotton fiber elongation. In this context, PA accumulation initiates cotton fiber thickening [97]. In Arabidopsis, PA interacts and suppresses the transport activity of AMMONIUM TRANSPORTER1 AMT1 leading to a reduced ammonium uptake, an important source of inorganic nitrogen. In *pldα1pldδ*-knockout double mutants of Arabidopsis, this was linked to an enhanced seedling growth under nitrogen deficiency [34]. Cumulatively, these results suggest that lipid signaling enzymes directly act in signaling, leading to plant acclimation to growth conditions and nutrient availability. 

## 4. Conclusions

It was decided to limit this article to focus on PLD and PI-PLC/DGK enzymes identified in crop species and acting in pathways leading to PA production. Of course, other enzymes also deserve to be studied with respect to crops. Phosphatidylinostol-4-kinases (PI4Ks) are the first enzymes that commit PI to the phosphoinositide pathway. PI4Ks lead to the formation of PI-4-P, which can produce PI-4,5-P_2_ when phosphorylated by phosphatidylinostol-4-phosphate-5-kinases. PI-4,5-P_2_ has an important role in defining the electrostatic properties of membranes, and it can tether proteins through specific domains [98]. The production and accumulation of PI-4,5-P_2_ is a characteristic of the plant response to salicylic acid [99]. Conversely, Arabidopsis plants mutated in some PI4Ks constitutively accumulate SA [100], while displaying altered auxin-related responses independently of SA accumulation [101,102].

Phospholipases A (PLA) produce lysophospholipids and free fatty acids from phospholipids, and a patatin-related phospholipase A (pPLAIIIα) was shown to play a complex role in Arabidopsis, affecting virus resistance, organ and seed size, as well as seed germination rate [103]. The hydrolysis of galactolipids by patatin-type phospholipases might also be involved in jasmonate synthesis [104].

Finally, lipid signaling in plants also includes signaling by phosphorylated sphingolipids [105]. Plant non-specific phospholipase C (NPC) can tentatively be an alternate DAG source for PA production by DGKs. The role of NPCs in regulating stress responses and physiological processes in crops is currently less studied compared to that of PI-PLC enzymes. In rice, OsNPC6 was shown to be involved in controlling mesocotyl elongation, an important process during plant germination [106]. Whether this effect is mediated by NPC-derived DAG entering PA production is yet to be shown.

In this article, structural features of PLD and PI-PLC/DGK enzymes were analyzed and discussed, leading to the conclusion that regulatory processes concerning these enzymes are likely conserved. Recent advances in the study of lipid signaling in crop plants have been summarized. Yet, to be able to show that enzymes of the lipid signaling pathways are involved in regulating a specific process, such as stress responses, nutrition, or development, it is necessary to block enzyme activities in planta, either via reverse genetic or pharmacological approaches. For crop species, it was, until recently, challenging to achieve this. The use of pharmacological agents is often limited to suspension cells and seedlings, whereas the use of mutants has been limited due to technical constraints. However, the genomes of many crop species are now available [107], and genome editing of crop plants has been recently made possible [108]. Presently, the CRISPR-Cas approach has been successfully implemented in many crops [109]. These advances will open up new avenues to study stress response lipid signaling in crop plants and thus allow us to understand the mechanisms employed by crop plants to acclimate to environmental stresses. This knowledge can then be used to generate plant lines with enhanced resilience by either genome editing or classical breeding. 

Finally, the identification of PA-binding proteins in crops appears to be the next research frontier. Structural and biochemical experiments will be necessary to cross it.

## 5. Materials and Methods

### 5.1. Sequence Alignment, Phylogenetic Analysis, and Consensus 

Sequences were retrieved from the UniProt databank. Our objective was to only consider the sequences annotated as phosphoinositide-dependent phospholipases C (EC:3.1.4.11), phospholipases D (EC:3.1.4.4), or diacylglycerol kinases (EC:2.7.1.107). The obtained list was curated this way: obsolete sequences were not considered; only sequences with an associated gene locus were considered. Possible false-positive sequences were identified because they were aberrant in the corresponding phylogenetic trees.

For RBOH proteins, the AtRBOHD sequence was used to blast the UniProt databank to search for orthologs. We considered the sequences with an identity higher than 24.5%. Only sequences with an associated gene locus were considered. Only one sequence per locus was considered.

Multiple sequence alignment was performed using the Clustal Omega webserver [110] with default settings were used. Clustal Omega uses the HHalign algorithm and its default settings as its core alignment engine. The algorithm is described in Söding, J. (2005) [111]. The default transition matrix is Gonnet, gap opening penalty is 6 bits, and gap extension is 1 bit.

Phylogenetic analysis of the sequences was conducted using the MEGA 11 software [112]. The phylogenetic tree was obtained using the maximum likelihood tree method with a bootstrap confidence cutoff of 50% based on 1000 random re-samplings (with replacement) of columns from the multiple sequence alignment.

The consensus logo motif was drawn by WebLogo [113]. Each residue in the alignment was assigned a color. No threshold for the presence of a residue on the column was applied. K, H or R is colored blue. P, A, I, L, M, F, W or V is colored black. E or D is colored red; N or Q is colored purple; Y, G, C, S or T is colored green. 

### 5.2. Prediction of Structures and Visualization of Structure

The structures were predicted by AlphaFold2 [37]. AlphaFold2 is a machine learning approach that incorporates physical and biological knowledge about protein structure, leveraging multi-sequence alignments, into the design of the deep learning algorithm. AlphaFold2 has combined numerous deep-learning innovations to predict the three-dimensional (3D) structures of proteins. It has been trained on protein chains from the PDB and uses the input sequence to query databases of protein sequences to construct a multiple-sequence alignment. The structures were retrieved through the AlphaFold2 Protein Structure Database [114]. The Alphafold2 structures can be colored based on the confidence with which each domain is modeled (Figure S5). 

Structures were represented by the PyMOL Molecular Graphics System, Version 2.0 Schrödinger, LLC [115]. 

## Data Availability

The original contributions presented in the study are included in the article/Supplementary Materials, further inquiries can be directed to the corresponding author.

## Supplementary Materials

The following supporting information can be downloaded at: https://www.mdpi.com/article/10.3390/plants13111532/s1, Figure S1: Predicted structure of TaPLD9; Figure S2: Differences in the C2 domain of α-subtype C2-PLDs versus non-α-subtype C2-PLDs; Figure S3: Structural features of the putative calcium-binding site in the catalytic site of PLDs; Figure S4: Structural features of the C2 domain of crop PI-PLCs; Figure S5: Predicted structures of TaPLD1, TaPLD9, TaPLD10, TaDGK1, TaDGK5, and TaPI-PLC2-1A. The structures were predicted by AlphaFold2. The color code corresponds to model confidence as defined by AlphaFold2. AlphaFold2 produces a per-residue confidence score, named “predicted local distance difference test” (pLDDT), that ranges between 0 and 100; Table S1: PLD proteins in a selection of model and crop plants; Table S2: DGK proteins in a selection of model and crop plants; Table S3: PI-LPC proteins in a selection of model and crop plants; Table S4: RESPIRATORY BURST OXIDASE HOMOLOGUEs (RBOHs) proteins in a selection of model and crop plants.
