# Supplementary material for: Phospholipid Signaling in Crop Plants: A Field to Explore
Source: Plants (Basel). 2024 May 31;13(11):1532. doi: 10.3390/plants13111532 (PMC11174929; doi:10.3390/plants13111532)
Supplement: Supplementary file 1 [file plants-13-01532-s001.zip › plants-2989582-supplementary/Supplementary_files/Supplementary_Figure_S1.pdf]

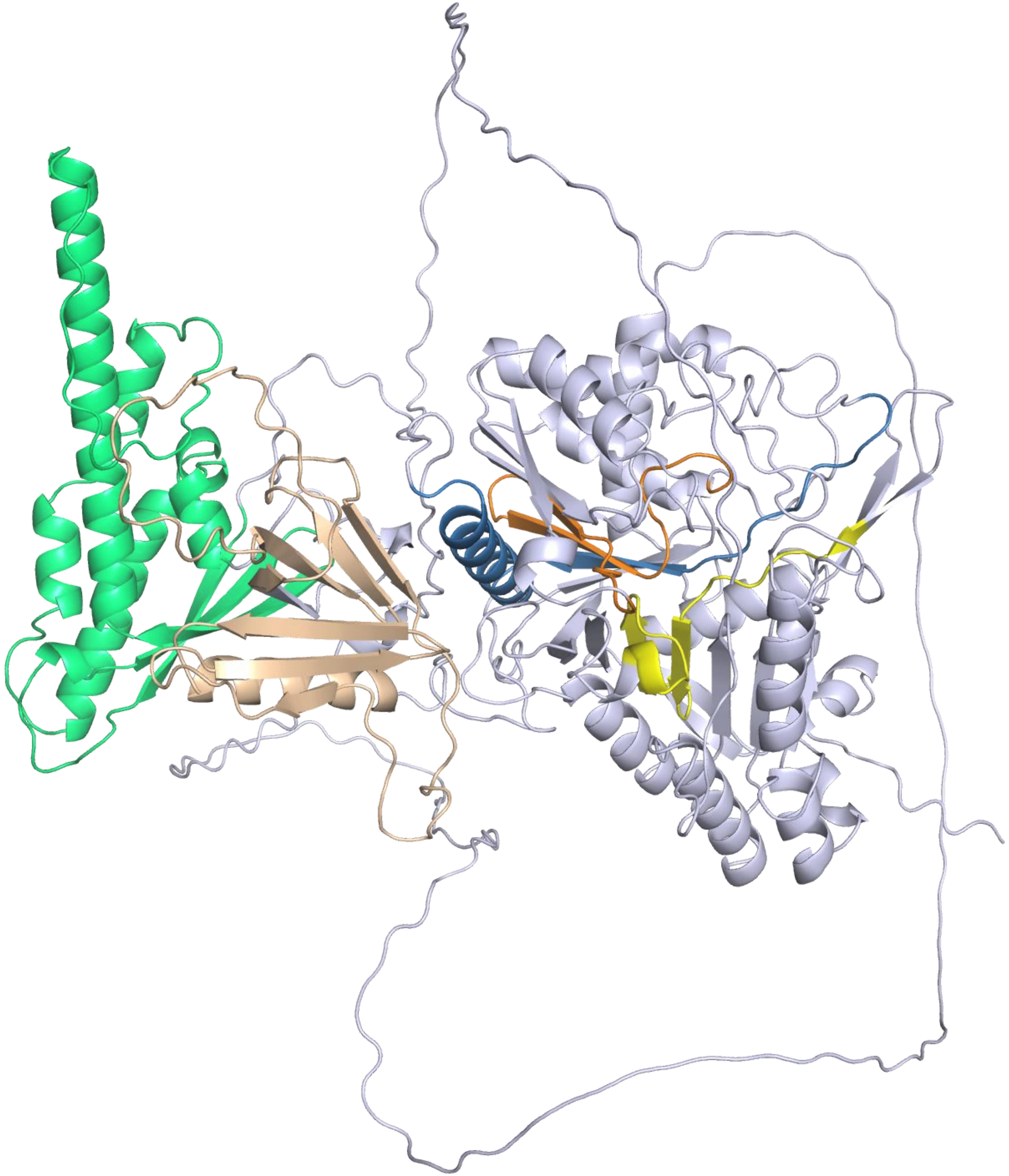

Figure S1. Predicted structure of TaPLD9. The structure was predicted by Alphafold [36] and represented by the PyMOL Molecular Graphics System, Version 2.0 Schrödinger, LLC. Green, PX domain; brown, PH domain; orange, first HKD domain; yellow, second HKD domain; red, PIP2 binding region 1 (PBR1, [47]) domain.
