# Supplementary material for: Phospholipid Signaling in Crop Plants: A Field to Explore
Source: Plants (Basel). 2024 May 31;13(11):1532. doi: 10.3390/plants13111532 (PMC11174929; doi:10.3390/plants13111532)
Supplement: Supplementary file 1 [file plants-13-01532-s001.zip › plants-2989582-supplementary/Supplementary_files/Supplementary_Figure_S2.pdf]

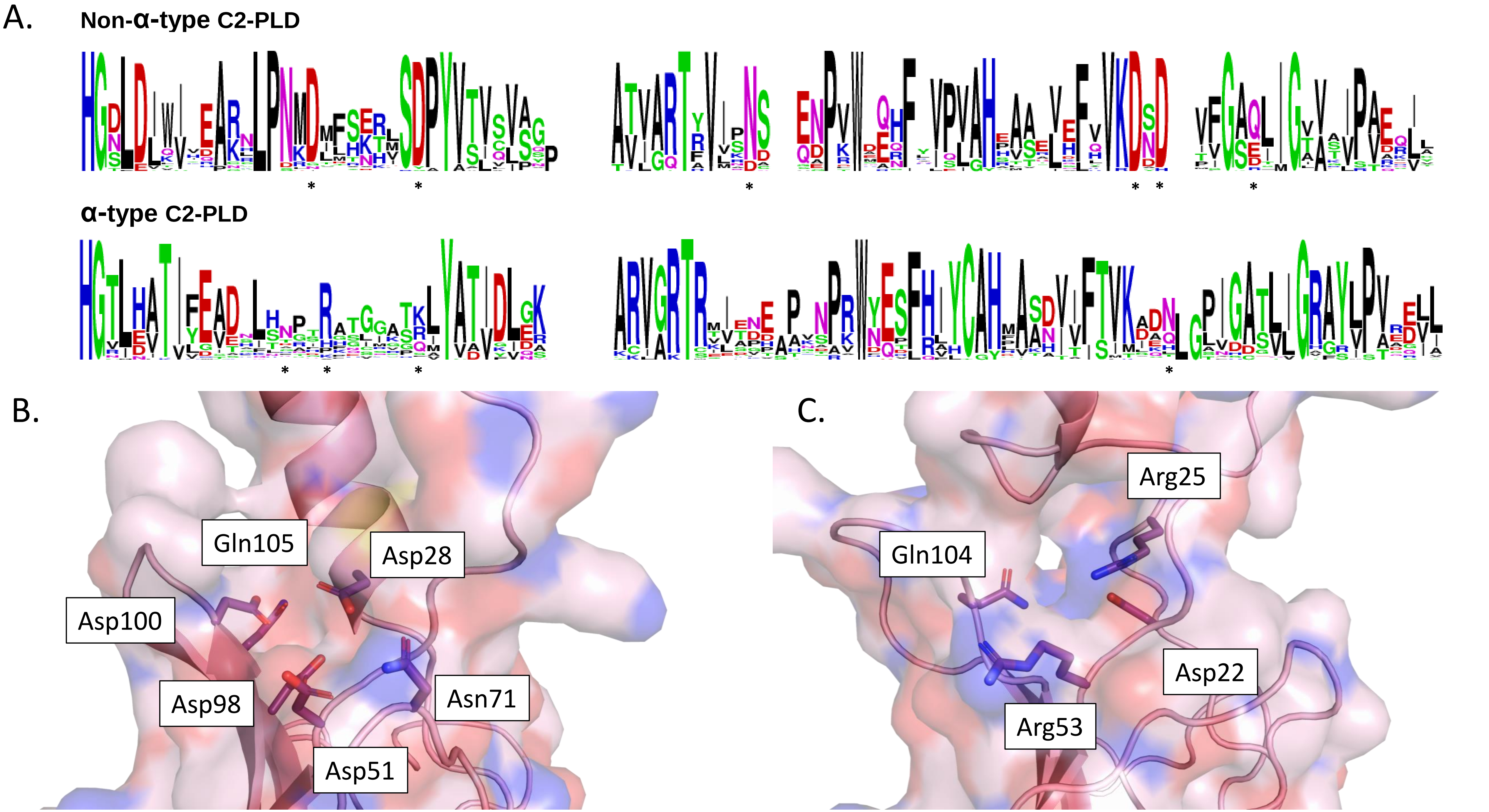

Figure S2. Differences in the C2 domain of α-type C2-PLDs versus non-α-type C2-PLDs. (**A**) Motif consensus of the C2 domains. Sequences of the C2 domains were retrieved and multiple sequence alignment was performed using the Clustal Omega webserver [45]. . The consensus logo motif was drawn by WebLogo [53] with the Clustal Omega Color code for the residues.The * indicates the residues shown in B and C. (**B**) Structure of the loop expected to coordinate calcium in the C2 domain of TaPLD8, a non-α-type C2-PLD. (**c**) Structure of the loop considered to coordinate calcium in TaPLD1, an α-type C2-PLD. The structures were predicted by Alphafold [36] and represented by the PyMOL Molecular Graphics System, Version 2.0 Schrödinger, LLC.
