# Supplementary material for: Phospholipid Signaling in Crop Plants: A Field to Explore
Source: Plants (Basel). 2024 May 31;13(11):1532. doi: 10.3390/plants13111532 (PMC11174929; doi:10.3390/plants13111532)
Supplement: Supplementary file 1 [file plants-13-01532-s001.zip › plants-2989582-supplementary/Supplementary_files/Supplementary_Figure_S3.pdf]

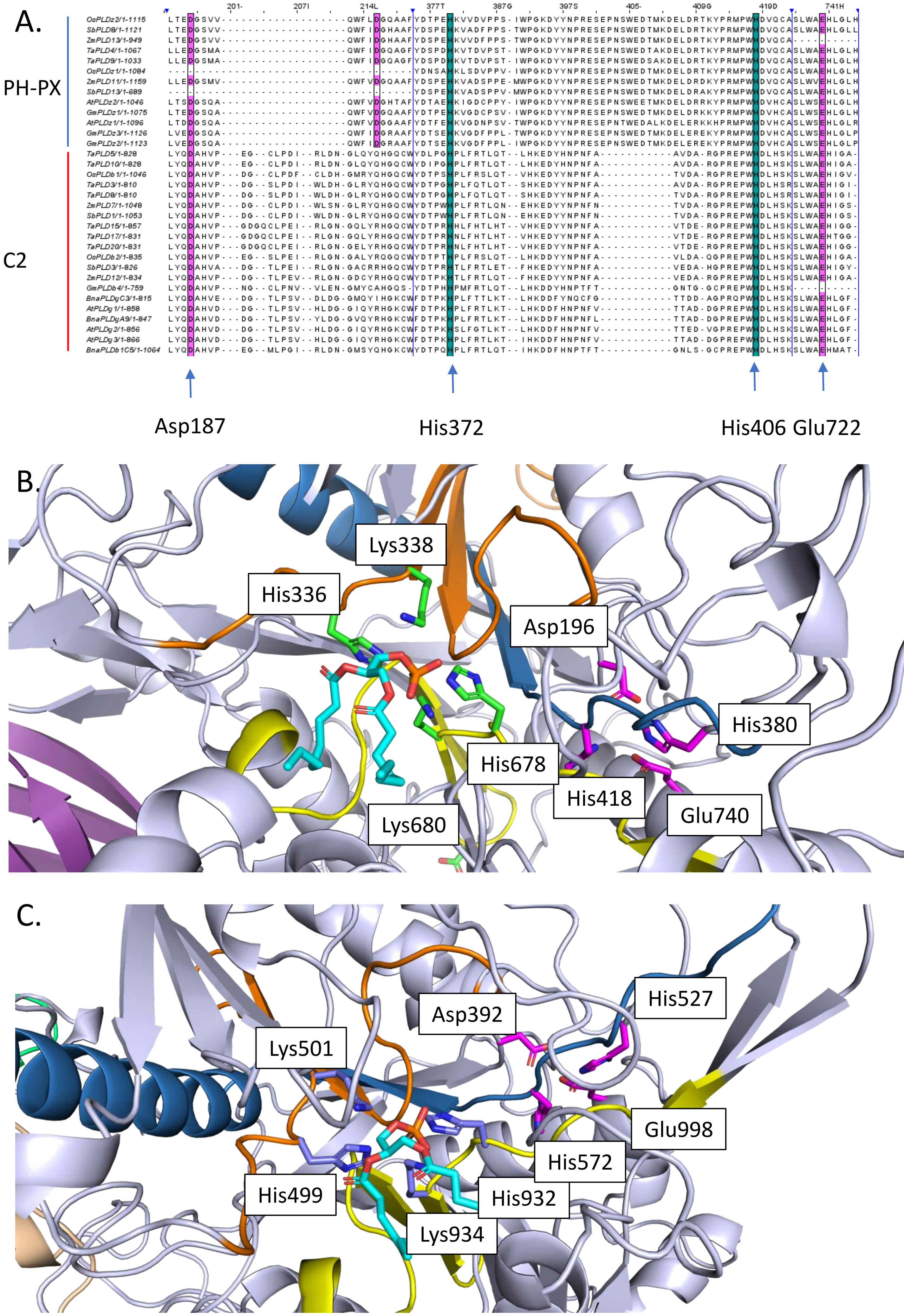

Figure S3. Structural features of the putative calcium binding site in the catalytic site of PLDs. (**A**) Alignment of the putative calcium binding residues in different plant PLDs. For PX-PH PLDs, the Asp (D) aligned with those in C2-PLDs in the primary sequences are not the ones that are aligned in the 3D structures; the Asp (D) aligned in the 3D structures are the Asp (D) 9 residues more C-terminal. The numbers refer to that in AtPLDα1. (**B**) Active site of TaPLD1(α-C2-PLD). Colors of the lateral chains: HKD (green) / Putative Ca2+ binding residues (magenta). The diC8-PA (cyan) was positioned by the structural alignment of TaPLD1 with PLDα1 the crystallized with diC8-PA. (**C**) Active site of TaPLD9 (PX-PH-PLD): Colours of the lateral chains: HKD (purple) / Putative Ca2+ binding residues (magenta). The diC8-PA (cyan) was positioned by the structural alignment of TaPLD9 with PLDα1 the crystallized with diC8-PA.
