# Supplementary material for: Phospholipid Signaling in Crop Plants: A Field to Explore
Source: Plants (Basel). 2024 May 31;13(11):1532. doi: 10.3390/plants13111532 (PMC11174929; doi:10.3390/plants13111532)
Supplement: Supplementary file 1 [file plants-13-01532-s001.zip › plants-2989582-supplementary/Supplementary_files/Supplementary_Figure_S4.pdf]

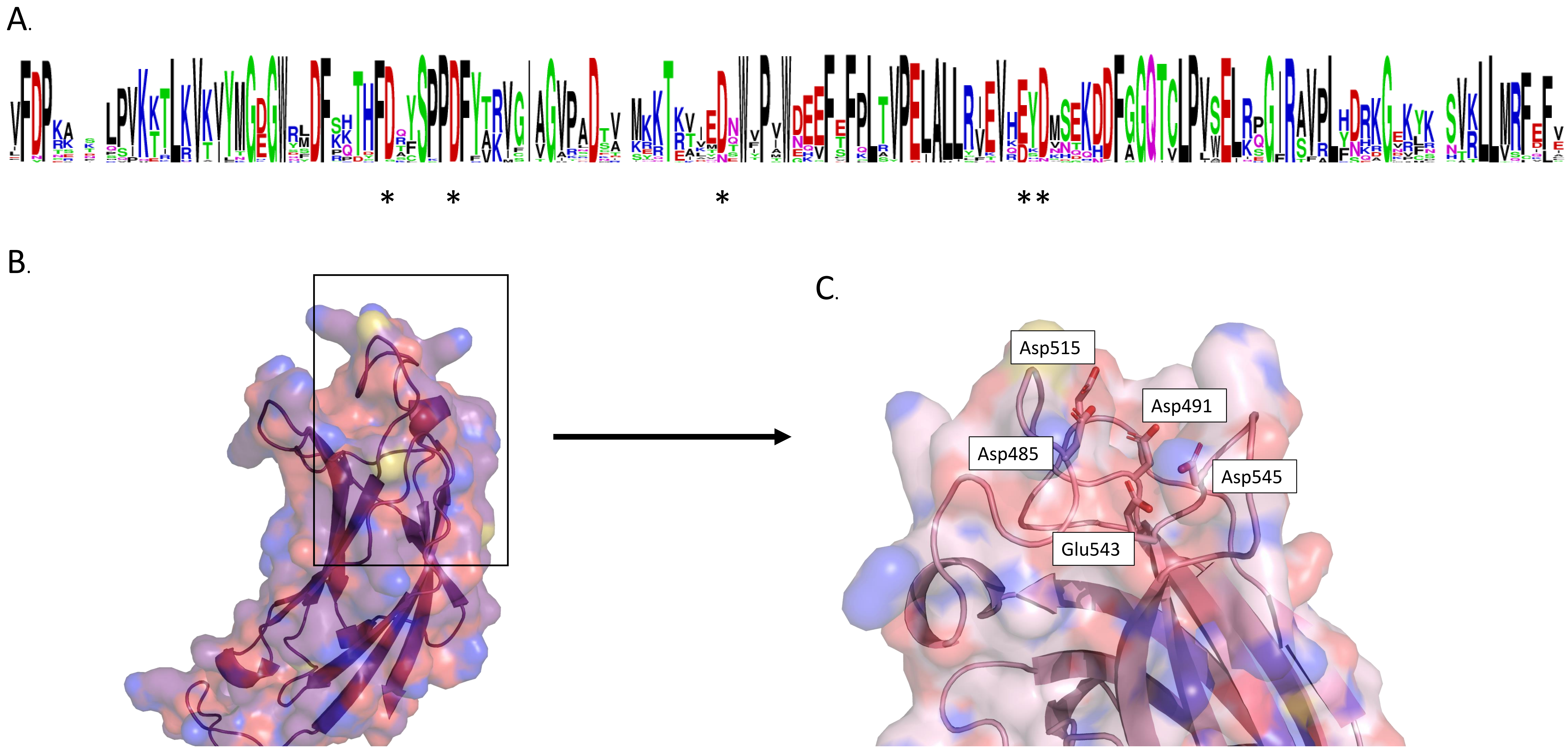

Figure 4. Structural features of the C2 domain of crop PI-PLCs. (**A**) Motif conservation of the catalytic residues. PI-PLC sequences of table S3 were aligned using the Clustal Omega webserver [45]. The consensus logo motif of the C2 domain was drawn by WebLogo [53] with the Clustal Omega Colorcode for the residues. The * indicate the residues displayed in B. (**B**) Structure of the C2 domain of TaPI-PLC2_1A. C. Close-up of the loop coordinating calcium. The structures were predicted by Alphafold [36] and represented by the PyMOL Molecular Graphics System, Version 2.0 Schrödinger, LLC.
