# Supplementary material for: Phospholipid Signaling in Crop Plants: A Field to Explore
Source: Plants (Basel). 2024 May 31;13(11):1532. doi: 10.3390/plants13111532 (PMC11174929; doi:10.3390/plants13111532)
Supplement: Supplementary file 1 [file plants-13-01532-s001.zip › plants-2989582-supplementary/Supplementary_files/Supplementary_Figure_S5.pptx]

## Slide 1
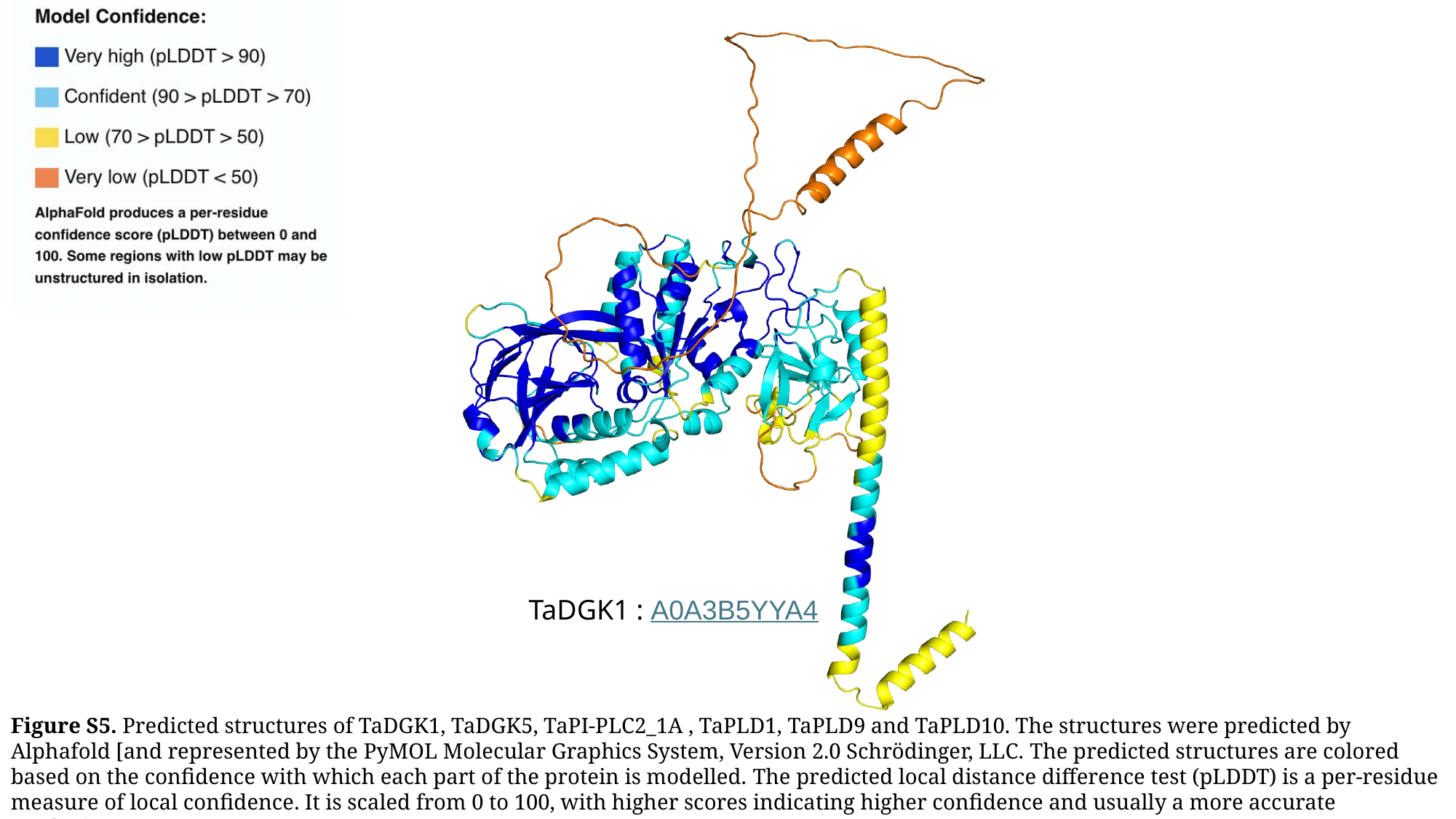

TaDGK1 : A0A3B5YYA4
Figure S5. Predicted structures of TaDGK1, TaDGK5, TaPI-PLC2_1A , TaPLD1, TaPLD9 and TaPLD10. The structures were predicted by Alphafold [and represented by the PyMOL Molecular Graphics System, Version 2.0 Schrödinger, LLC. The predicted structures are colored based on the confidence with which each part of the protein is modelled. The predicted local distance difference test (pLDDT) is a per-residue measure of local confidence. It is scaled from 0 to 100, with higher scores indicating higher confidence and usually a more accurate prediction.

## Slide 2
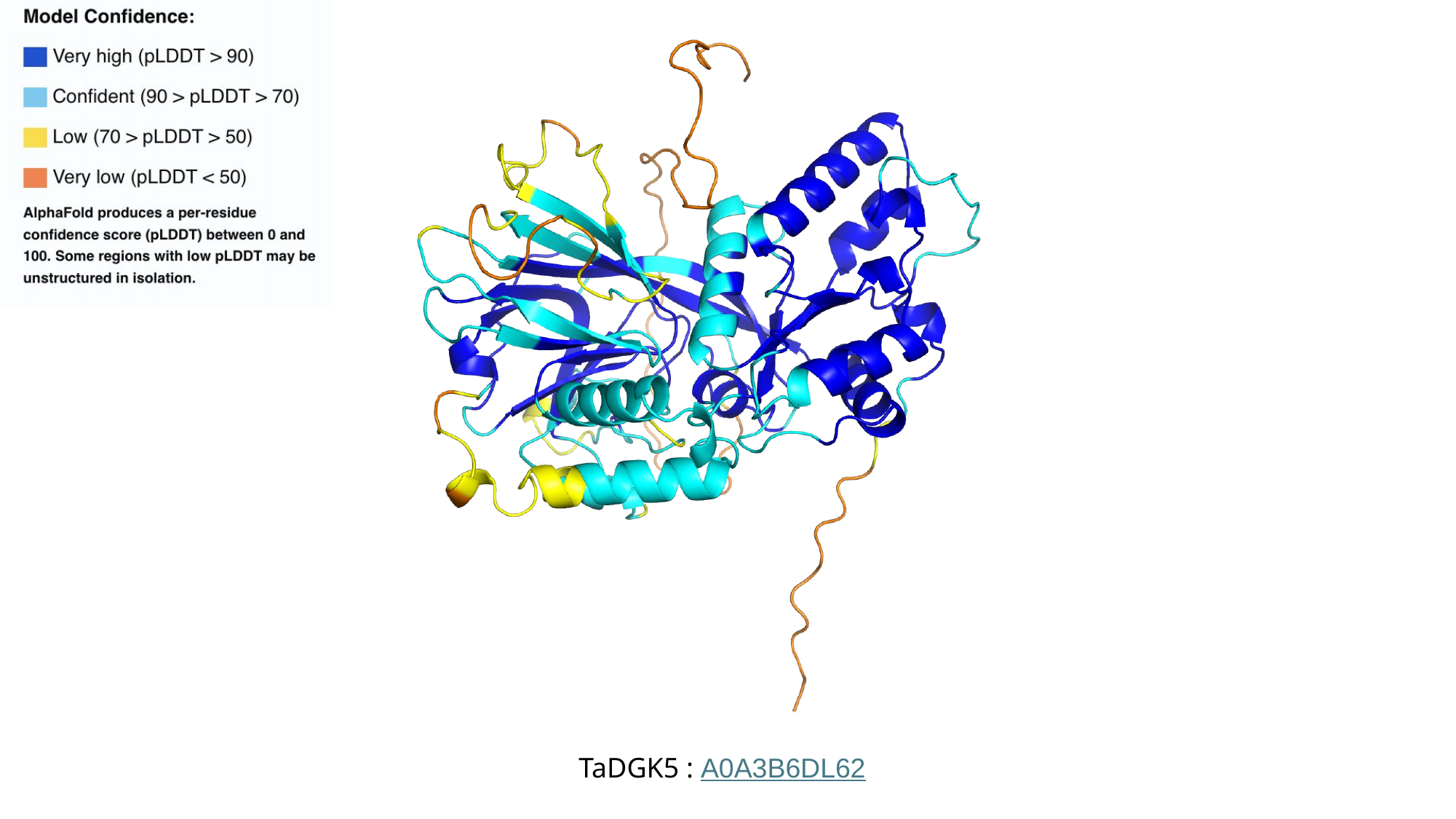

TaDGK5 : A0A3B6DL62

## Slide 3
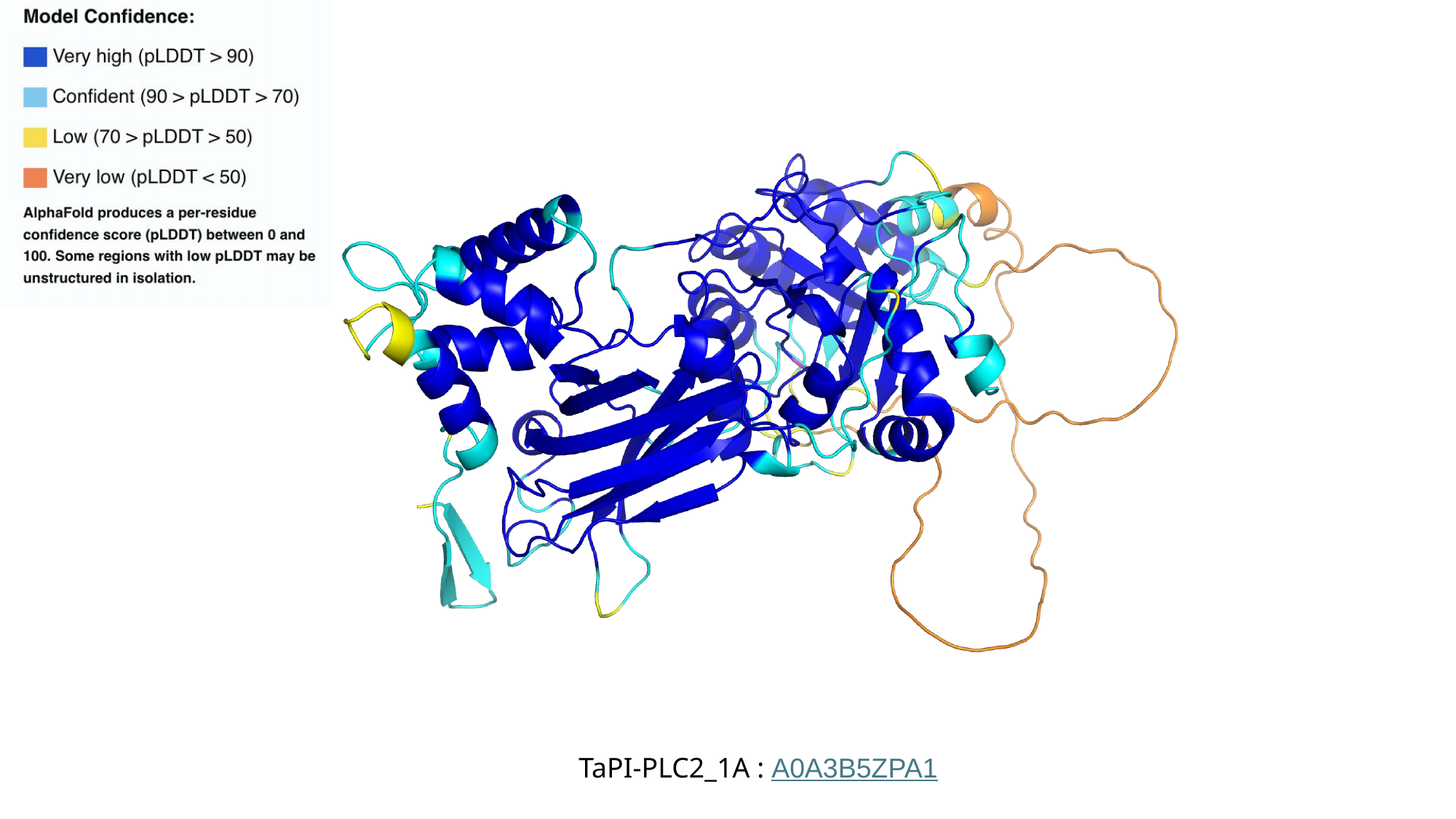

TaPI-PLC2_1A : A0A3B5ZPA1

## Slide 4
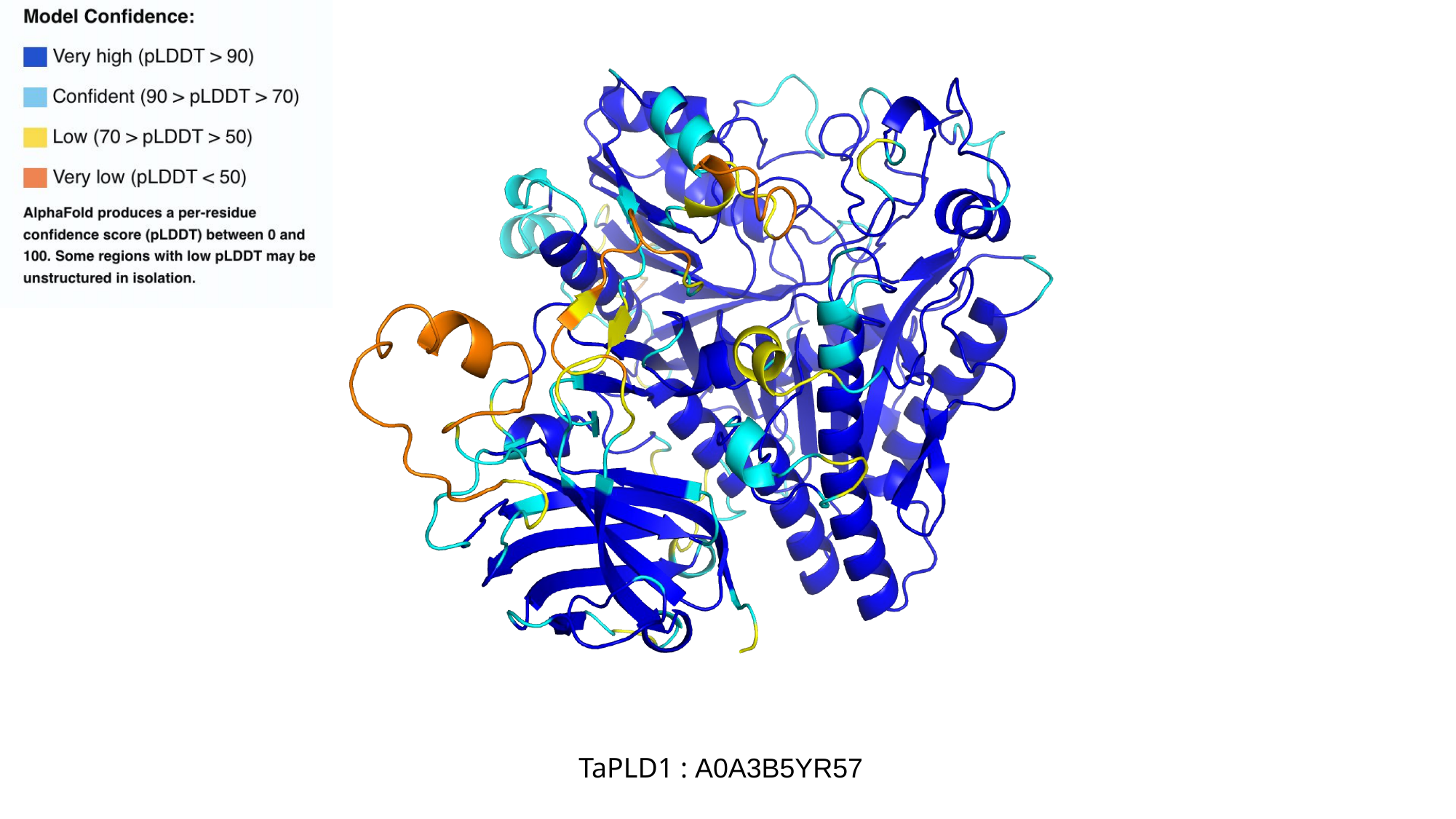

TaPLD1 : A0A3B5YR57

## Slide 5
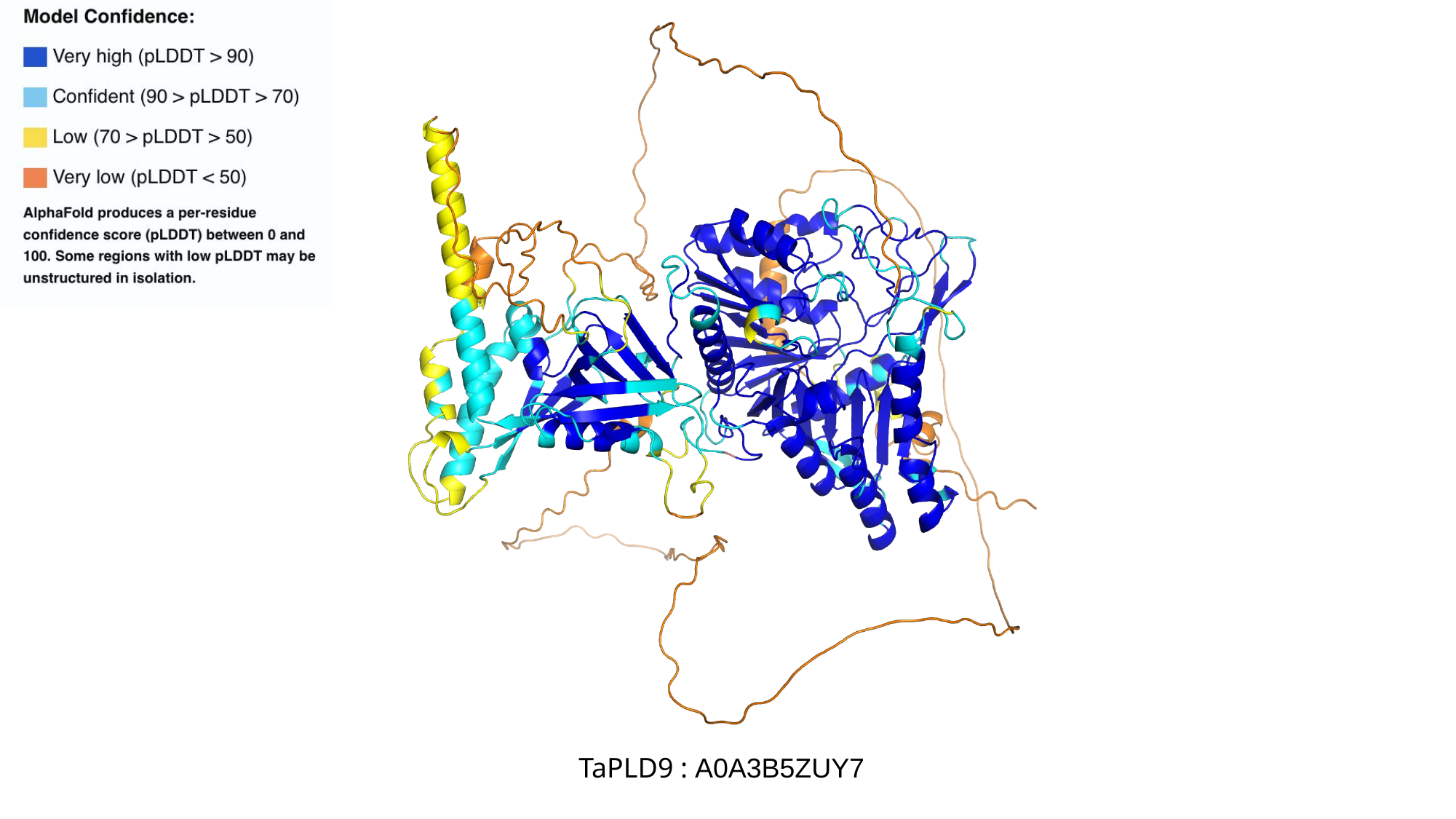

TaPLD9 : A0A3B5ZUY7

## Slide 6
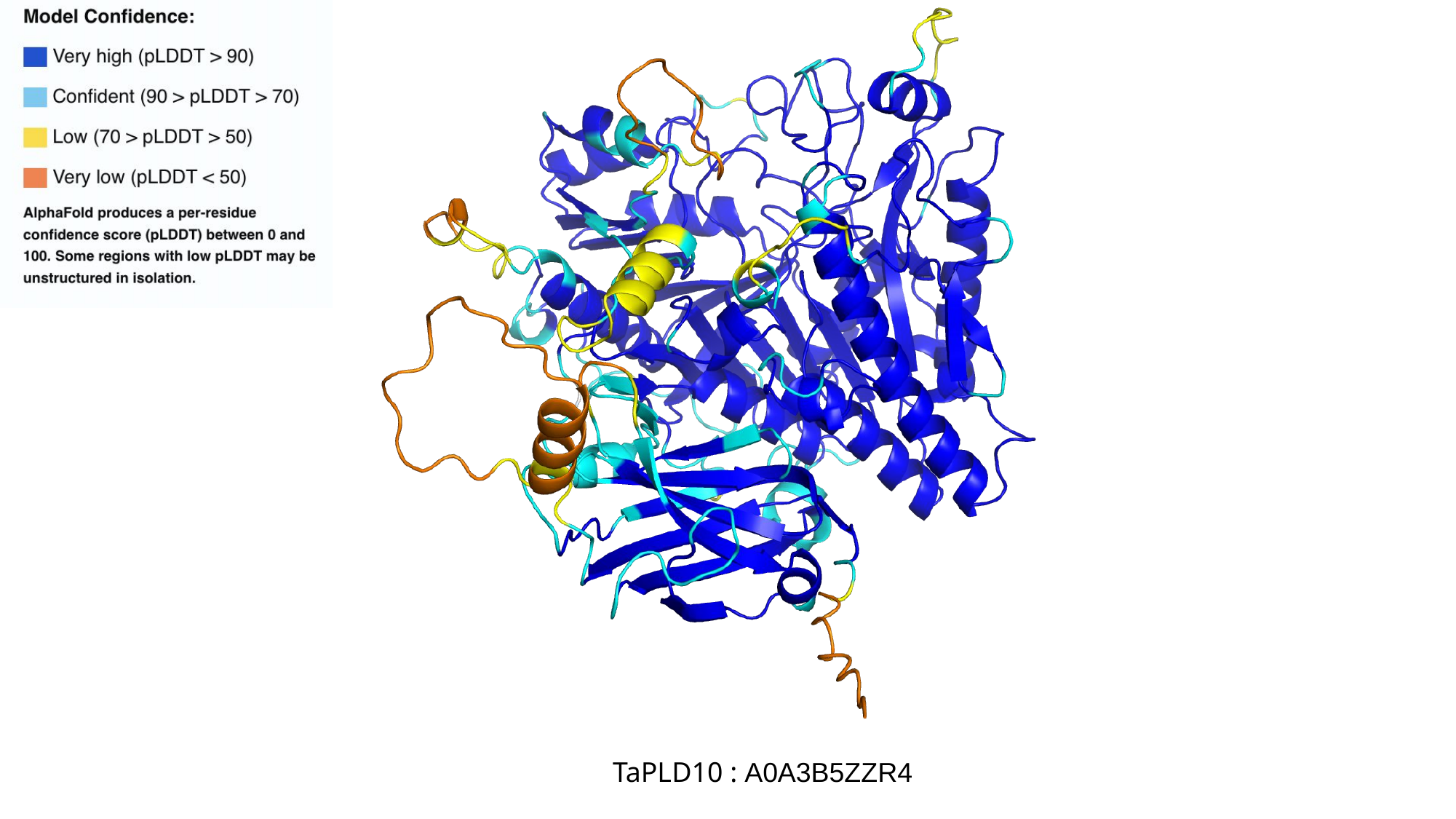

TaPLD10 : A0A3B5ZZR4
