# Supplementary material for: Phospholipid Signaling in Crop Plants: A Field to Explore
Source: Plants (Basel). 2024 May 31;13(11):1532. doi: 10.3390/plants13111532 (PMC11174929; doi:10.3390/plants13111532)
Supplement: Supplementary file 1 [file plants-13-01532-s001.zip › plants-2989582-supplementary/Supplementary_files/Table S1.pdf]

Table S1. PLD proteins in a selection of model and crop plants. Protein sequences were retrieved from UniProtKB (The Uniprot Consortium, 2023). We selected only sequences that were associated with a gene or locus. Protein names are based on the name given in UniProtKB (Arabidopsis thaliana) or names given in [37-40] for *Zea mays*, *Oryza sativa*, for *Brassica napus* and *Glycine max* PLDs. For *Zea mays*, *Triticum aestivum*, *Sorghum bicolor* and *Solanum tuberosum* protein names were given by us. Sequences considered as Obsolete in UniProtKB were not considered. This leads to less sequences than considered in other publications (for instance 18 PLDs for *Brassica napus* versus 32 in [39]. This is a table. Tables should be placed in the main text near to the first time they are cited.

Species	Gene name	Gene index/locus	Protein ID
Arabidopsis thaliana	AtPLDα1	At3g15730	Q38882
	AtPLDα2	At1G52570	Q9SSQ9
	AtPLDα3	At5g25370	P58766
	AtPLDα4	At1g55180	Q9C888
	AtPLDβ1	At2g42010	P93733
	AtPLDβ2	At4g00240	O23078
	AtPLDδ	At4g35790	Q9C5Y0
	AtPLDγ1	At4g11850	Q9T053
	AtPLDγ2	At4g11830	Q9T051
	AtPLDγ3	At4g11840	Q9T052
	AtPLDζ1	At3g16785	Q9LRZ5
	AtPLDζ2	At3g05630	Q9M9W8
Brassica napus	BnaPLDα1A1	BnaA01g28530D	A0A816Y1E3
	BnaPLDα1A5	BnaA05g23740D	A0A078G696
	BnaPLDα1C1	BnaC01g35830D	A0A078G5Y0
	BnaPLDα1C5	BnaC05g37540D	A0A078GZU4
	BnaPLDα2A5	BnaA05g35700D	A0A078J6Z0
	BnaPLDα2C3	BnaC03g69220D	A0A078I9G0
	BnaPLDβ1C5	BnaC05g00230D	A0A078FQJ8
	BnaPLDβ2Ann	BnaAnng13600D	A0A078IUA4
	BnaPLDβ2Cnn	BnaCnng23890D	A0A078IT23
	BnaPLDγC3	BnaC03g73050D	A0A078JCN5
	BnaPLDγA9	BnaA09g21320D	UPI0004EEE1AF
	BnaPLDδA8	BnaA08g04540D	A0A078HG45
	BnaPLDδAnn	BnaAnng08250D	A0A078IA90
	BnaPLDδC3	BnaC03g62540D	A0A078HSW7
	BnaPLDδC7	BnaC07g45790D	A0A816NDM4
	BnaPLDδCnn	BnaCnng54530D	A0A078JLP4
	BnaPLDεA6	BnaA06g00490D	A0A078GFB2
	BnaPLDεC6	BnaC06g07140D	A0A078H387
Oryza sativa	*OsPLDα1*	Os01g0172400	Q43007
	OsPLDα2	Os05g0171000	Q65XR9
	OsPLDα3	Os06g0604400	P93844
	OsPLDα4	Os06g0604200	Q69X22
	OsPLDα5	Os06g0604300	Q69X21
	OsPLDα6	Os03g0391400	B9F8T8
	OsPLDα7	Os08g0401800	Q0J5U3
	OsPLDα8	Os09g0421300	Q0J1P8
	OsPLDβ1	Os10g0524400	Q0IW93

	OsPLDβ2	Os03g0119100	Q0DVP7
	OsPLDδ1	Os09g0543100	Q0IZX5
	OsPLDδ2	Os03g0840800	Q0DLX5
	OsPLDδ3	Os07g0260400	Q0D7D5
	OsPLDζ1	Os05g0358700	Q6L479
	OsPLDζ2	Os01g0310100	Q7F0T4
Zea mays	ZmPLD1	Zm00001d039670	A0A804MZ94
	ZmPLD2	Zm00001d008727	K7VQG5
	ZmPLD3	Zm00001d037643	A0A1D6LZK1
	ZmPLD4	Zm00001d046508	B8A045
	ZmPLD5	Zm00001d029397	A0A096T2N3
	ZmPLD6	Zm00001d005770	A0A1D6EQ16
	ZmPLD7	Zm00001d029684	A0A1D6K719
	ZmPLD8	Zm00001d006257	A0A1D6EU73
	ZmPLD9	Zm00001d022594	A0A1D6IPN3
	ZmPLD10	Zm00001d005484	A0A1D6ENA4
	ZmPLD11	Zm00001d037946	A0A1D6M211
	ZmPLD12	Zm00001d027386	A0A1D6JLG0
	ZmPLD13	Zm00001d009075	A0A1D6FHM8
	ZmPLD14	Zm00001d015033	A0A1D6GYU0
Triticum aestivum	TaPLD1	CFC21_005124	A0A3B5YR57
	TaPLD2	CFC21_006035	A0A3B5YV71
	TaPLD3	CFC21_006664	A0A3B5YWT0
	TaPLD4	CFC21_007124	A0A3B5YZ63
	TaPLD5	CFC21_008554	A0A3B5Z2S1
	TaPLD6	CFC21_008657	A0A3B5Z4I2
	TaPLD7	CFC21_010006	A0A3B5ZN33
	TaPLD8	CFC21_011612	A0A3B5ZTI3
	TaPLD9	CFC21_012022	A0A3B5ZUY7
	TaPLD10	CFC21_013447	A0A3B5ZZR4
	TaPLD11	CFC21_029396	A0A3B6A1V1
	TaPLD12	CFC21_040433	W5D4Q6
	TaPLD13	CFC21_046449	A0A3B6GQ57
	TaPLD14	CFC21_054247	A0A3B6HZE6
	TaPLD15	CFC21_059471	A0A3B6IVQ4
	TaPLD16	CFC21_059750	A0A3B6IXW5
	TaPLD17	CFC21_063501	A0A3B6JPL0
	TaPLD18	CFC21_065980	A0A3B6KGE5
	TaPLD19	CFC21_067134	A0A3B6KNI0
	TaPLD20	CFC21_069027	A0A3B6KRX6
	TaPLD21	CFC21_071541	A0A3B6LM60
	TaPLD22	CFC21_071762	A0A3B6LMM1
	TaPLD23	CFC21_072953	A0A3B6LQ66
	TaPLD24	CFC21_074856	A0A3B6LWC0
	TaPLD25	CFC21_077548	A0A3B6MSR3
	TaPLD26	CFC21_077775	A0A3B6MS95
	TaPLD27	CFC21_078943	A0A3B6MXD3

	TaPLD28	CFC21_080850	A0A3B6N0P7
	TaPLD29	CFC21_082094	A0A3B6NKG8
	TaPLD30	CFC21_086727	A0A3B6PF94
	TaPLD31	CFC21_100643	A0A3B6RP28
	TaPLD32	CFC21_100644	A0A3B6RSP8
	TaPLD33	CFC21_103911	A0A3B6SN50
	TaPLD34	CFC21_105912	A0A3B6SV41
	TaPLD35	CFC21_110202	A0A3B6TN85
Sorghum bicolor	SbPLD1	SORBI_3001G320200	C5WUK8
	SbPLD2	SORBI_3001G349800	A0A1B6QMR6
	SbPLD3	SORBI_3001G529800	A0A1B6QQY3
	SbPLD4	SORBI_3002G098900	C5X2U3
	SbPLD5	SORBI_3002G204500	C5XCW2
	SbPLD6	SORBI_3002G282500	A0A1B6QDV2
	SbPLD7	SORBI_3003G050400	A0A1B6Q1F1
	SbPLD8	SORBI_3003G147000	A0A1B6Q391
	SbPLD9	SORBI_3004G016900	A0A1Z5RKN2
	SbPLD10	SORBI_3005G222500	C5Y8F2
	SbPLD11	SORBI_3008G183400	C5YSV6
	SbPLD12	SORBI_3009G062600	A0A1Z5R170
	SbPLD13	SORBI_3009G109925	A0A1Z5R234
	SbPLD14	SORBI_3010G185600	C5Z5N7
Solanum tuberosum	StPLD1	102588687	M1AKN6
	StPLD2	102591907	M1AD93
	StPLD3	102595135	M1CS26
	StPLD4	102597936	M0ZIV2
	StPLD5	102600860	M1C2T7
Glycine max	GmPLDα1	GLYMA_08G211700	A0A0R0IQ92
	GmPLDα2	GLYMA_07G031100	I1KH24
	GmPLDα3	GLYMA_13G364900	I1M5T2
	GmPLDβ1	GLYMA_18G288600	I1N548
	GmPLDβ2	GLYMA_02G093500	I1JDQ3
	GmPLDβ3	GLYMA_07G080400	I1KIJ3
	GmPLDβ4	GLYMA_03G018900	K7KCA2
	GmPLDδ1	GLYMA_11G081500	I1LI58
	GmPLDδ2	GLYMA_01G162100	I1J8G3
	GmPLDδ3	GLYMA_05G168300	I1K497
	GmPLDδ4	GLYMA_06G020500	I1K7F8
	GmPLDδ5	GLYMA_04G020400	I1JSY8
	GmPLDγ	GLYMA_01G215100	I1JA23
	GmPLDε1	GLYMA_07G010900	I1KGD7
	GmPLDε2	GLYMA_15G023500	I1MCY6
	GmPLDζ1	GLYMA_20G238000	I1NJ37
	GmPLDζ2	GLYMA_15G152100	I1MGP5
	GmPLDζ3	GLYMA_09G041400	I1L0W3
