# Supplementary material for: Phospholipid Signaling in Crop Plants: A Field to Explore
Source: Plants (Basel). 2024 May 31;13(11):1532. doi: 10.3390/plants13111532 (PMC11174929; doi:10.3390/plants13111532)
Supplement: Supplementary file 1 [file plants-13-01532-s001.zip › plants-2989582-supplementary/Supplementary_files/Table S2.pdf]

Table S2. DGK proteins in a selection of model and crop plants. Protein sequences were retrieved from UniProtKB (The Uniprot Consortium, 2023). We selected only sequences that were associated with an ORF or a locus. Protein names are based on the name given in UniProtKB (*Arabidopsis thaliana),* or names given in [64,65] for *Brassica napus.* and *Glycine max*. For *Oryza sativa, Zea mays, Triticum aestivum*, *Sorghum bicolor* and *Solanum tuberosum* protein names were given by us. Sequences considered as Obsolete in UniProtKB were not considered. This leads to less sequences than considered in other publications (for instance 15 DGKs for *Brassica napus* versus 32 in [64]).

Species	Gene name	Gene index/locus	Protein ID
Arabidopsis thaliana	*AtDGK1*	At5G07920	Q39017
	AtDGK2	At5G63770	Q9FFN7
	AtDGK3	At2G18730	Q8VZG1
	AtDGK4	At5G57690	Q1PDI2
	AtDGK5	At2G20900	Q9C5E5
	AtDGK6	At4g28130	F4JKI3
	AtDGK7	At4g30340	F4JQ95
Brassica napus	BnaDGK2-2	BnaC02g42690D	A0A078HRB4
	BnaDGK2-3	BnaA06g22880D	A0A078HSL2
	BnaDGK3-1	BnaA09g09730D	A0A078GYS5
	BnaDGK3-2	BnaC09g51050D	A0A078IKB8
	BnaDGK3-3	BnaC07g03080D	A0A078FJ59
	BnaDGK3-4	BnaA07g01550D	A0A078IH63
	BnaDGK4-1	BnaC03g71550D	A0A078JNT0
	BnaDGK4-2	BnaA03g10150D	A0A816V8H1
	BnaDGK5-2	BnaA09g43530D	A0A078FL38
	BnaDGK5-3	BnaC09g10500D	A0A816IS55
	BnaDGK5-4	BnaA09g10340D	A0A078HPK0
	BnaDGK6-1	BnaA03g49040D	A0A078GZ62
	BnaDGK6-2	BnaC07g50560D	A0A078J458
	BnaDGK7-1	BnaA01g06700D	A0A078GSK6
	BnaDGK7-2	BnaC01g08060D	A0A816R188
Oryza sativa	*OsDGK1*	Os01g0783200	A0A0P0V8Z9
	OsDGK2	Os02g0787800	A0A0P0VQI3
	OsDGK3	Os03g0425300	Q75GS0
	OsDGK4	Os04g0634700	Q7XQT2
	OsDGK5	Os08g0178700	A0A0P0XCH7
	OsDGK6	Os12g0576900	Q2QN70
Zea mays	ZmDGK1	Zm00001d029550	A0A1D6K5X0
	ZmDGK2	Zm00001d024212	A8QM11
	ZmDGK3	Zm00001d024192	A0A1D6IXW6
	ZmDGK4	Zm00001d002163	C0P2P0
	ZmDGK5	Zm00001d018608	A0A1D6HQL7
	ZmDGK6	Zm00014a_029504	A0A3L6E0U9
	ZmDGK5	Zm00001d023979	C0PCE8
	ZmDGK6	Zm00001d030770	A8QM12
	ZmDGK9	Zm00001d033807	A0A1D6L2J7

	ZmDGK7	Zm00001d043263	A0A1D6N9T9
	ZmDGK8	Zm00014a_001719	A0A3L6FGS5
	ZmDGK9	Zm00014a_002902	A0A3L6G4C6
	ZmDGK10	Zm00014a_008345	A0A8J8YCR8
	ZmDGK11	Zm00014a_014987	A0A3L6FXN1
	ZmDGK12	Zm00014a_016979	A0A3L6G7A0
	ZmDGK13	Zm00014a_025605	A0A3L6G881
	ZmDGK14	Zm00014a_027339	A0A317Y3W3
	ZmDGK18	Zm00014a_038728	A0A3L6FV25
	ZmDGK15	Zm00014a_041790	A0A317YID7
Triticum aestivum	TaDGK1	CFC21_007197	A0A3B5YYA4
	TaDGK2	CFC21_012079	A0A3B5ZV78
	TaDGK3	CFC21_019787	A0A3B6B7Y5
	TaDGK4	CFC21_026196	A0A3B6CHW0
	TaDGK5	CFC21_032494	A0A3B6DL62
	TaDGK6	CFC21_036442	A0A3B6EKJ0
	TaDGK7	CFC21_042470	A0A3B6FRQ6
	TaDGK8	CFC21_048335	A0A3B6GX87
	TaDGK9	CFC21_064531	A0A3B6KB32
	TaDGK10	CFC21_064833	A0A3B6KEP3
	TaDGK11	CFC21_064833	A0A3B6KFG2
	TaDGK11	CFC21_070308	A0A3B6LFN3
	TaDGK12	CFC21_070599	A0A3B6LIB9
	TaDGK13	CFC21_076649	A0A3B6MLU9
	TaDGK14	CFC21_084891	A0A3B6NTN4
	TaDGK15	CFC21_089681	A0A3B6PS22
	TaDGK16	CFC21_090155	A0A3B6PUV0
	TaDGK17	CFC21_094139	A0A3B6QMP0
	TaDGK18	CFC21_095073	A0A3B6MM22
	TaDGK19	CFC21_098017	A0A3B6RGI4
	TaDGK20	CFC21_103035	A0A3B6SDT2
	TaDGK21	CFC21_104075	A0A3B6SK59
	TaDGK22	CFC21_109363	A0A9R1MKJ3
	TaDGK23	CFC21_110342	A0A3B6TVF0
Sorghum bicolor	*SbDGK1*	SORBI_3001G333900	C5WWI1
	SbDGK2	SORBI_3003G318700	C5XLR4
	SbDGK3	SORBI_3004G324100	C5XUA5
	SbDGK4	SORBI_3006G230400	C5YGP3
	SbDGK5	SORBI_3007G062000	C5YHX6
	SbDGK6	SORBI_3007G091400	A0A1B6PGN0
	SbDGK7	SORBI_3008G077600	A0A1B6PC38
	SbDGK8	SORBI_3008G140400	A0A1B6PDT2
Solanum tuberosum	*StDGK1*	102598468	M1CA25
	StDGK2	102603370	M1C4Y6
Glycine max	*GmDGK1*	Glyma.13G302200	A0A0R0GW00
	GmDGK2	Glyma.05G196100	I1K533

GmDGK3	Glyma.12G200100	I1LUC0
GmDGK4	Glyma.06G299200	K7KY74
GmDGK5	Glyma.06G223900	K7KWM3
GmDGK8	Glyma.06G254900	A0A368UI28
GmDGK9	Glyma.12G146700	A0A0R0HEZ8
GmDGK10	Glyma.17G067400	I1MSV0
GmDGK11	Glyma.17G077100	I1MT53
GmDGK12	Glyma.05G022500	I1JZK2
