# Supplementary material for: Phospholipid Signaling in Crop Plants: A Field to Explore
Source: Plants (Basel). 2024 May 31;13(11):1532. doi: 10.3390/plants13111532 (PMC11174929; doi:10.3390/plants13111532)
Supplement: Supplementary file 1 [file plants-13-01532-s001.zip › plants-2989582-supplementary/Supplementary_files/Table S3.pdf]

Table S3. PI-PLC proteins in a selection of model and crop plants. Protein sequences were retrieved from UniProtKB (The Uniprot Consortium, 2023). We selected only sequences that were associated with an ORF or a locus. Protein names are based on the name given in UniProtKB (*Arabidopsis thaliana)*. *Brassica napus* we numbered the sequences and indicated the correspondence with the names given in [75] for. For Glycine max, *Oryza sativa*, *Zea mays*, *Triticum aestivum*, *Sorghum bicolor* and *Solanum tuberosum* protein names were given by us. Sequences considered as Obsolete in UniProtKB were not considered. This leads to less sequences than considered in other publications. * indicates that in the alignment of PLC-X and PLC-Y regions some gaps are present that does not allow to verify the conservation of catalytic residues for these sequences.

Species	Gene name	Gene index/locus	Protein ID
	AtPLC1	At5g58670	Q39032
	AtPLC2	At3g08510	Q39033
	AtPLC3	At4g38530	Q56W08
	AtPLC4	At5g58700	Q944C1
Arabidopsis thaliana	AtPLC5	At5g58690	Q944C2
	AtPLC6	At2g40116	Q8GV43
	AtPLC7	At3g55940	Q9LY51
	AtPLC8	At3g47290	Q9STZ3
	AtPLC9	At3g47220	Q6NMA7
	BnPLC1/BnaPI-PLC4A2	BnaA02g07530D	A0A078H4B8
	BnPLC2/BnPI-PLC1A2 *	BnaA02g07560D	A0A078H5G4
	BnPLC3	BnaA03g18880D	A0A078F7J1
	BnPLC4/BnaPI-PLC2A5	BnaA05g29670D	A0A078HGK7
	BnPLC5/BnaPI-PLC6A3	BnaA06g40820D	A0A078JVP1
	BnPLC6 *	BnaA06g40830D	A0A078JBM3
	BnPLC7/BnaPI-PLC7A9	BnaA09g35480D	A0A078GPU6
	BnPLC8/BnaPI-PLC1A10	BnaA10g29490D	A0A078IM21
	BnPLC9/BnaPI-PLC5A10	BnaA10g29500D	A0A078IQC5
	BnPLC10/BnaPI-PLC4A10	BnaA10g29510D	A0A078IQE1
	BnPLC11/BnaPI-PLC1Ann-1	BnaAnng17370D	A0A078J897
Brassica napus	BnPLC12 *	BnaAnng32110D	A0A078JWK4
	BnPLC13/BnaPI-PLC1Ann-2	BnaAnng36640D	A0A078JUK0
	BnPLC14/BnaPI-PLC2C1	BnaC01g39480D	A0A078GBT7
	BnPLC15/BnaPI-PLC6C3	BnaC03g72360D	A0A078J6Y9
	BnPLC16/BnaPI-PLC6C4	BnaC04g05240D	A0A078G1Z6
	BnPLC17	BnaC04g56690D	A0A078JBC1
	BnPLC18/BnaPI-PLC2C5	BnaC05g44000D	A0A078GTM6
	BnPLC19/BnaCnng14930D	BnaCnng14930D	A0A078IBZ2
	BnPLC20 *	BnaCnng61000D	A0A078JTX5
	BnPLC21/BnaPI-PLC7Cnn-1	BnaCnng67650D	A0A078JU50
	BnPLC22/BnaPI-PLC4Unn	BnaUnng04440D	A0A078JXW9
	OsPLC1	Os03g0289300	B9F7R4
	OsPLC2 *	Os03g18000	Q10MY6
	OsPLC3	Os03g18010	Q10MY5
Oryza mays	OsPLC4	Os05g0127200	A0A0P0WHG1
	OsPLC5	Os07g0694000	Q6Z3Y9
	OsPLC6 *	Os12g0562400	Q0IML8
	OsPLC7	Os12g37560	Q2QNK1
	ZmPLC1	Zm00001d007229	A0A1D6F512
Zea mays	ZmPLC2	Zm00001d014903	A0A1D6GXH6
	ZmPLC3	Zm00001d014906	A0A1D6GXI3

	ZmPLC4	Zm00001d028746	A0A1D6JZ59
	ZmPLC5	Zm00001d047447	K7VZB3
Triticum aestivum	TaPI-PLC2-1A	CFC21_010388	A0A3B5ZPA1
	TaPI-PLC1-2A	CFC21_015202	A0A3B6AR20
	TaPI-PLC3-4A	CFC21_051891	A0A3B6HQB3
	TaPI-PLC4-5A	CFC21_065324	A0A3B6KDX2
	TaPI-PLC4-5D	CFC21_077049	A0A3B6MPW1
	TaPI-PLC1-2D	CFC21_027821	A0A3B6D6L9
	TaPI-PLC3-4D	CFC21_061978	A0A3B6JJX5
Sorghum bicolor	Sb_PIPLC1	SORBI_3001G408001	A0A1Z5SA06
	Sb_PIPLC2	SORBI_3002G430500	C5X6G9
	Sb_PIPLC3	SORBI_3005G002000	A0A1Z5RG00
	Sb_PIPLC4	SORBI_3005G018900	A0A1Z5RHC6
	Sb_PIPLC5	SORBI_3008G002000	C5YQ01
	Sb_PIPLC6	SORBI_3009G027800	A0A1Z5R1B1
Solanum tuberosum	StPLC1	102603424	M1AVA1
Glycine max	GmPLC1 *	GLYMA_02G226800	A0A0R0L9Z2
	GmPLC2	GLYMA_02G257000	I1JI88
	GmPLC3	GLYMA_02G257100	I1JI89
	GmPLC4	GLYMA_02G257200	I1JI90
	GmPLC5 *	GLYMA_11G229900	A0A0R0HUL4
	GmPLC6	GLYMA_11G230000	K7LRR0
	GmPLC7	GLYMA_11G230100	I1LMR9
	GmPLC8	GLYMA_14G059200	Q43443
	GmPLC9	GLYMA_14G059400	A0A0R0GLL7
	GmPLC10	GLYMA_14G193800	I1MBB2
	GmPLC11	GLYMA_18G027100	A0A0R0F6Q0
	GmPLC12	GLYMA_18G027200	K7MPJ5
	GmPLC13	GLYMA_18G027300	I1MZ23
