# Supplementary material for: Phospholipid Signaling in Crop Plants: A Field to Explore
Source: Plants (Basel). 2024 May 31;13(11):1532. doi: 10.3390/plants13111532 (PMC11174929; doi:10.3390/plants13111532)
Supplement: Supplementary file 1 [file plants-13-01532-s001.zip › plants-2989582-supplementary/Supplementary_files/Table S4.pdf]

Table S4. RESPIRATORY BURST OXIDASE HOMOLOGUEs (RBOHs) proteins in a selection of model and crop plants. Protein sequences were retrieved from UniProtKB (The Uniprot Consortium, 2023). We selected only sequences that were associated with an ORF or a locus. Protein names are based on the name given in UniProtKB. Sequences considered as Obsolete in UniProtKB were not considered. This leads to less sequences than considered in other publications.

Species	Gene name	Gene index/locus	Protein ID
Arabidopsis thaliana	AtRBOHD	AT5G47910	Q9FIJ0
Brassica napus	BnRBOHD-1.1		A0A2R4QM88
Oryza mays	OsRBOHB	Os01g0360200	Q5ZAJ0
Zea mays	ZmRBOHD	Zm00014a_029430	A0A3L6FZ80
Triticum aestivum	TaRbohI	CFC21_063272	A0A3B6JNW9
	TaRbohH	CFC21_012626	A0A3B5ZXM0
Glycine max		GLYMA_17G078300	I1MT65
		GLYMA_05G021100	K7KME9
